# Ectodermal loss of ARHGAP29 alters epithelial morphology and disrupts murine palatogenesis

**DOI:** 10.1242/dev.205636

**Published:** 2026-06-29

**Authors:** Emily Adelizzi, Lindsey Rhea, Campbell Mitvalsky, Samuel Pek, Bethany Doolittle, Martine Dunnwald

**Affiliations:** ^1^ Department of Anatomy and Cell Biology, The University of Iowa, 51 Newton Road, Iowa City, IA 52245, USA; ^2^ Interdisciplinary Graduate Program in Genetics, The University of Iowa, 51 Newton Road, Iowa City, IA 52245, USA

**Keywords:** Palatogenesis, Mouse, ARHGAP29, Development, Cleft

## Abstract

Orofacial clefts, including cleft palate (CP), are among the most common types of birth defects. CP results from a failure of palatal shelf fusion during development. Previous human studies have shown that variants of the RhoA GTPase activating protein 29 (ARHGAP29) gene are linked to CP, yet the role and tissue-specific requirements for ARHGAP29 during palatogenesis remain unknown. Here, we use tissue-specific deletion of *Arhgap29* in mice to provide the first direct evidence that ARHGAP29 is essential for palatal elevation and fusion. We demonstrate that ectodermal conditional loss of *Arhgap29* induces a significant delay in palatogenesis at embryonic day (E) 14.5 and a significant, complete cleft of the secondary palate at E18.5, which is not observed when *Arhgap29* is lost later in development using K14-Cre and K6-Cre. Phenotypic analyses of palatal shelves at E14.5 reveal a disorganized and thicker epithelium. Loss of *Arhgap29* increases palatal epithelial cell area and upregulates markers of contractility including alpha-smooth muscle actin and phospho-myosin regulatory light chain, implicating cell morphology and contractility as potential drivers of CP.

## Teaser

**Summary:** Tissue-specific deletion of *Arhgap29* in mice reveals that ectodermal ARHGAP29 is essential for proper palatal elevation and fusion, and that loss of *Arhgap29* alters oral epithelial morphology and upregulates contractility proteins.

## INTRODUCTION

Orofacial clefts (OFCs) are among the most common type of birth defects in humans, affecting roughly 1 in every 700 live births (Dixon et al., 2011). One type of OFC, non-syndromic (NS) cleft lip with or without cleft palate (CL/P), exhibits a complex etiology with both genetic and environmental underpinnings contributing to the defect (Rahimov et al., 2012). One particularly well-defined gene regulatory network contributing to NSCL/P is the interferon regulatory factor 6 (IRF6) gene regulatory network (Leslie et al., 2012). Mutations and single-nucleotide polymorphisms in *IRF6* cause and are associated with syndromic CL/P and NSCL/P, respectively (Kondo et al., 2002; Zucchero et al., 2004). In addition to this direct role for *IRF6* in CL/P, genome-wide association and exome sequencing studies have revealed that variants of downstream effector genes, such as RhoA GTPase activating protein 29 (*ARHGAP29*), are associated with and causative of CL/P, yet these studies are lacking functional evidence for a role of ARHGAP29 in CL/P (Beaty et al., 2010; Leslie et al., 2012; Liu et al., 2017; Savastano et al., 2017).

Palatogenesis is the embryonic process by which the secondary palate forms. Starting at embryonic day (E) 12.5 in the mouse, the walls of the maxillary processes undergo bilateral growth, giving rise to two palatal shelves. Palatal shelves comprise a mesenchymal core containing neural crest-derived mesenchymal cells underlying a layer of ectoderm-derived oral epithelial cells, themselves covered by a single layer of specialized squamous epithelial cells termed periderm (Hammond et al., 2019; Whittaker and Adams, 1971). These palatal shelves grow vertically alongside the tongue (E13.5) and remodel to a horizontal position above the tongue by E14.0. They subsequently grow towards each other and the ectoderm-derived cells [forming the so-called medial edge epithelium (MEE)] covering the mesenchyme begin undergoing major morphogenic events; first, they make contact, then the superficial periderm is removed, followed by the intercalation of epithelial cells into a single cell layer (called the medial epithelial seam; E14.5), and finally they are removed as the palate forms (E15.0). This removal leads to the formation of a confluent mesenchymal bridge on the roof of the mouth, formally giving rise to the separate oral and nasal cavities (Bush and Jiang, 2012; Ferguson, 1988, 1995; Lan et al., 2015). Palatal fusion is not uniform across the antero-posterior axis; instead, the palatal shelves first fuse in the middle and then fusion zippers in the anterior and posterior directions. Following fusion of the palatal shelves, the secondary palate fuses anteriorly with the primary palate, starting at the incisive foramen. When the palatal shelves fail to fuse, this results in a cleft palate, which is lethal in the mouse as the animals cannot suckle and feed at birth. Loss of many genes causes cleft palate in the mouse, including those coding for transcription factors (e.g. Irf6, Trp63) (Ingraham et al., 2006; Leoyklang et al., 2006; Thomason et al., 2008), growth factors (e.g. Tgfb3, Fgf10), cell adhesion proteins (e.g. Cdh1, Nectin4) (Alvizi et al., 2023; Lough et al., 2020; Tomlinson et al., 2012) and cytoskeletal proteins (e.g. Specc1l, Ctnnb1) (Hall et al., 2020; Saadi et al., 2023; Wang et al., 2023), many of which belong to or are influenced by the RhoA pathway, suggesting that cytoskeletal remodeling may be driving palatogenesis.

RhoA is a small GTPase that shuttles between an active and an inactive form based on its binding to GTP or GDP, respectively (Ibrahim and Richman, 2026). Guanine nucleotide exchange factors (GEFs), guanine nucleotide dissociation factors (GDIs) and GTPase activating proteins (GAPs) regulate this activity (Müller and Goody, 2018; Siderovski and Willard, 2005). Over 60 GAPs have been identified; among them, the PTPL1-associated RhoGAP1 (PARG1, reclassified as ARHGAP29) was described as a specific inhibitor of RhoA signaling with a strong expression in human heart and skeletal muscle tissues (Saras et al., 1997). Subsequent studies found ARHGAP29 in many other human and murine tissues and cell types, including endothelial cells, keratinocytes, and to a lesser degree fibroblasts (Leslie et al., 2012; Paul et al., 2017; Rhea et al., 2025; Xu et al., 2011). Most of the initial work on ARHGAP29 was done in endothelial cells, where it reduces RhoA activity at cell–cell adhesion sites to promote their remodeling during tubulogenesis (Post et al., 2013; Tagashira et al., 2018; Xu et al., 2011). Subsequent studies have found that ARHGAP29 contributes to the definition of apical-lateral border polarity in epithelial tight junctions (Tan et al., 2020) and participates in the specification of the non-neuronal ectoderm fate (Collier et al., 2023). In cutaneous keratinocytes, it is required for cell shape, proliferation and migration (Rhea et al., 2025), while during cancer metastasis ARHGAP29 is upregulated and mediates Yes-associated protein 1 (YAP1)-dependent actin remodeling in response to tissue stiffness (Kolb et al., 2020; Qiao et al., 2017; Shimizu et al., 2020). Collectively, these studies demonstrate the importance of ARHGAP29 in various developmental events. This is further supported by the findings that *Arhgap29^fl^*;*Sox2-Cre* and *Arhgap29*-deficient murine embryos die prior to chorioallantoic fusion (before E8.5) (Barry et al., 2016; Paul et al., 2017; Xu et al., 2011). We previously identified several *ARHGAP29* genetic variants in individuals with CL/P (Leslie et al., 2012; Liu et al., 2017) and subsequently developed a murine model with a point mutation that mimics an *ARHGAP29* variant (K326X) found in an individual with CL/P (Paul et al., 2017). This single-nucleotide, nonsense mutation produces a truncated protein that is presumed to lack function because no homozygous *Arhgap29^K326X^* offspring were observed (as early as E8.5). While heterozygosity for this allele did not result in CL/P, it did cause intra-oral adhesions, a well-accepted hallmark of cleft palate. However, due to the early lethality of the global *Arhgap29* knockouts, these experiments did not shed light on the function of ARHGAP29 during orofacial morphogenesis. Finally, a recent report describes CRISPR-based *Arhgap29* knockout animals surviving a few days after birth, although details on ARHGAP29 levels in the craniofacial region are lacking (Chi et al., 2025; Zhang et al., 2025). Collectively, these studies provide evidence for a role, yet undefined, for ARHGAP29 in palatogenesis. They also highlight the limitations of our current models and underline the need for new alleles that can overcome early embryonic lethality.

Prior whole-mount and tissue *in situ* expression analyses show that the *Arhgap29* transcript is detected throughout embryonic craniofacial structures, including the palatal shelves (Leslie et al., 2012). Detailed immunofluorescence analysis revealed the presence of the ARHGAP29 protein in the mesenchyme, oral epithelium, and periderm of the palatal shelves, with particularly high expression in ectoderm-derived cells (Leslie et al., 2012). However, it is unclear which tissues require ARHGAP29 to promote proper palatogenesis and what the function of ARHGAP29 is during this process. This study is the first tissue-specific removal of *Arhgap29* used to investigate the mechanisms and requirement for ARHGAP29 during palatogenesis in the mouse. It reveals a role for ARHGAP29 in controlling the morphology and organization of epithelial cells covering the palatal shelves and in inhibiting markers of contractility, including phosphorylated myosin regulatory light chain (pMRLC) and alpha-smooth muscle actin (α-SMA), in a spatial and temporal manner during palatogenesis.

## RESULTS

### Generation and validation of *Arhgap29* conditional knockouts

**Fig. 1.**
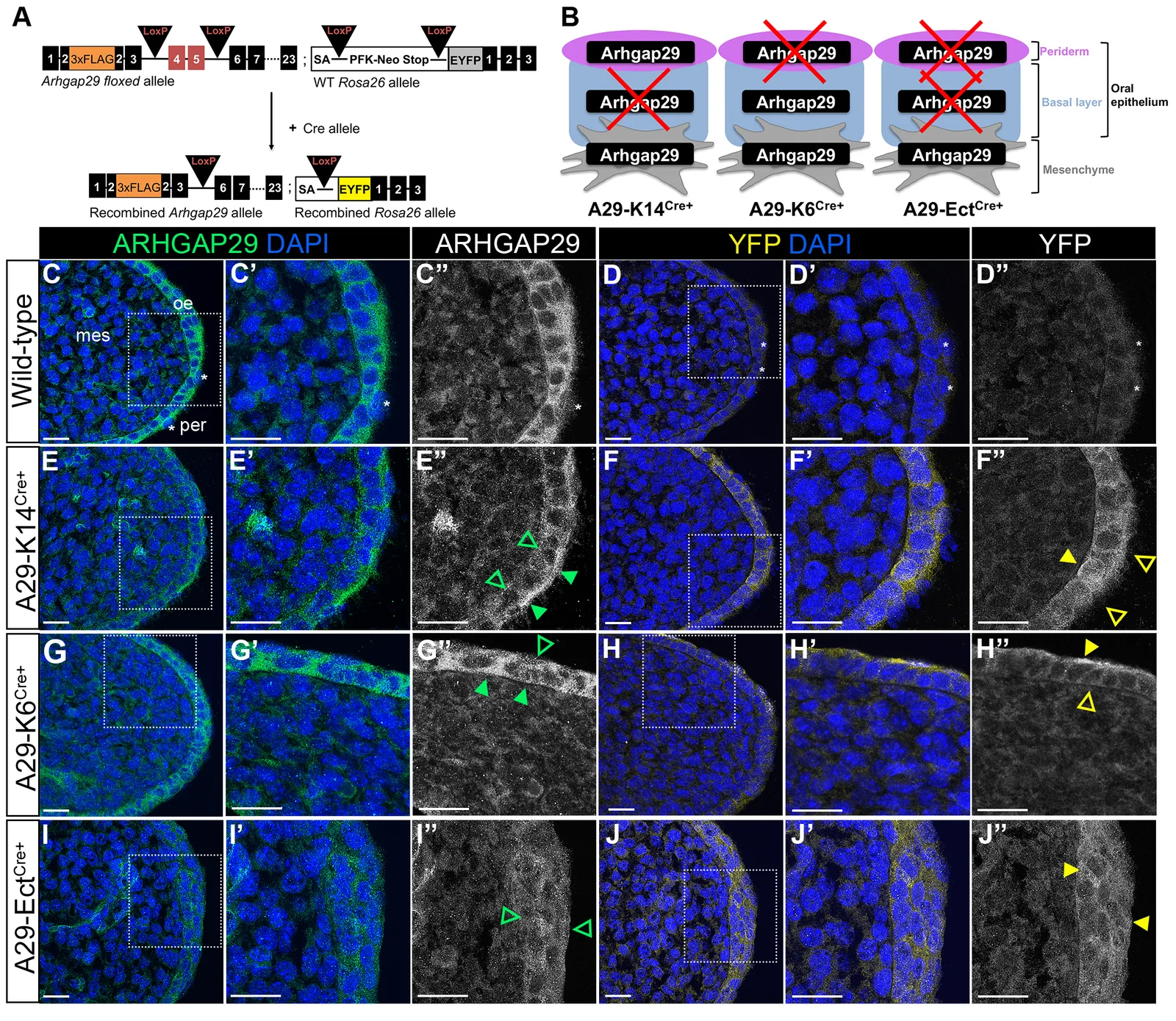
**Validation of *Arhgap29* conditional knockouts.** (A) Top: Schematic of 3xFLAG-tagged *Arhgap29* floxed allele and *Rosa26* allele harboring a YFP reporter. Bottom: Upon Cre recombination, the LoxP sites of the *Arhgap29* allele and the *Rosa26* allele are excised, resulting in a truncated *Arhgap29* gene product and YFP expression, respectively. (B) Schematic of *Arhgap29* knockout in (from left to right): *Arhgap29^fl/fl^;K14^Cre^*^+^ (A29-K14^Cre+^), *Arhgap29^fl/fl^;K6^Cre^*^+^ (A29-K6^Cre+^) and *Arhgap29^fl/fl^;Ect^Cre+^* (A29-Ect^Cre+^) murine models. *Arhgap29* is removed only in basal epithelial cells in the *Arhgap29^fl/fl^;K14^Cre^*^+^, only in the periderm cells in the *Arhgap29^fl/fl^;K6^Cre+^*, and in the entire oral epithelium (basal and periderm cells) in the *Arhgap29^fl/fl^;Ect^Cre+^*. (C-J″) Maximum projections of two to four confocal slices of E14.5 elevated palatal shelf stained for ARHGAP29 (green in merged) and YFP (yellow in merged) of wild-type (C-D″), *Arhgap29^fl/fl^;K14^Cre^*^+^ (E-F″), *Arhgap29^fl/fl^;K6^Cre+^* (G-H″), and *Arhgap29^fl/fl^;Ect^Cre+^* (I-J″) embryos; boxed regions are shown at higher magnification on the right. Solid and open arrowheads indicate cells lacking or expressing ARHGAP29 (green) or YFP (yellow), respectively. Asterisks denote periderm cells. mes, mesenchyme; oe, oral epithelium; per, periderm. Scale bars: 20 μm.

ARHGAP29 is detected throughout the oral cavity, including the oral epithelium and periderm, both of which are ectoderm derived. To evaluate the cell-specific requirements of *Arhgap29* during palatogenesis, we used an established *Arhgap29* floxed (*Arhgap29^fl^*) allele (Barry et al., 2016) and crossed it with a 129S4.Cg-*E2f1>Tg(Wnt1-cre)2Sor*/J (abbreviated *Wnt1^Cre^*) driver to generate neural crest cell-specific knockout (Fig. S1A,B). We modified this *Arhgap29^fl^* allele using CRISPR to knock in 3xFLAG in exon 2 immediately following the translation start site and crossed this FLAG-tagged *Arhgap29^fl^* allele with either a *Tg(KRT14-cre)1Efu* (abbreviated *K14^Cre^*) (Vasioukhin and Fuchs, 2001), a *Krt6a^em2(icre/ERT2)Vk^* (abbreviated *K6^Cre^*) (Saroya et al., 2023) or a *Tg(Tcfap2a-cre)1Will* (abbreviated *Ect^Cre^*) (Forni et al., 2011; Schock et al., 2017) driver to generate epithelial only, periderm only, or ectoderm (epithelial and periderm) specific knockouts, respectively (Fig. 1A,B). Both *Arhgap29^fl^* alleles contain LoxP sites flanking exons 4 and 5. These two exons are excised upon Cre recombination resulting in the production of a truncated nonfunctional ARHGAP29 protein that is targeted for degradation (Barry et al., 2016). We validated the recombination of *Arhgap29^fl^* by PCR for all alleles, by ARHGAP29 immunofluorescence only for the *Wnt1^Cre^* (Fig. S1C-D′), and utilized an additional YFP reporter allele that is activated upon any Cre recombination for all other Cre drivers (Fig. 1A). Immunofluorescence analysis of wild-type (WT) E14.5 embryos showed strong expression of ARHGAP29 in ectoderm-derived tissues (oral epithelium and periderm) and concomitant absence of YFP in these same tissues (Fig. 1C-D″). ARHGAP29 was specifically reduced in only the basal epithelial cells of *Arhgap29^fl/fl^;K14^Cre+^* embryos, and not in the superficial periderm cells, while YFP was only detected in basal epithelial cells (Fig. 1E-F″). In *Arhgap29^fl/fl^;K6^Cre+^* embryos, a reduction of ARHGAP29 in the periderm was difficult to detect (Fig. 1G-G″) but YFP showed specific expression in the periderm (Fig. 1H-H″), supporting successful recombination in this layer. Conversely, we found a reduction of ARHGAP29 in all ectoderm-derived cells of *Arhgap29^fl/fl^;Ect^Cre+^* embryos, including in periderm cells (Fig. 1I-I″). Similarly, YFP was detected in both epithelial and periderm cells (Fig. 1J-J″). These data demonstrate the effective recombination of the *Arhgap29^fl^* allele in the intended and distinct ectoderm-derived cell populations. Of note, all *Arhgap29^fl/fl^;Ect^Cre+^* mutants showed some degree of YFP expression in the underlying mesenchyme (Fig. S2), suggesting some recombination of the *Arhgap29^fl^* allele in mesenchymal cells either inherited from germline recombination or through ectopic Cre expression. Consequently, the *Arhgap29^fl/fl^;Ect^Cre+^* should be considered a combined mesenchyme knockdown/ectoderm knockout rather than a strict ectoderm-specific conditional murine model, but will still be referred to as *Arhgap29^fl/fl^;Ect^Cre+^* throughout this article.

### ARHGAP29 is not required in the mesenchyme for proper palatogenesis

Given the off-target reduction of *Arhgap29* in the mesenchyme observed in *Arhgap29^fl/fl^;Ect^Cre+^* mutants, we examined whether a reduction of ARHGAP29 in the mesenchyme alone would impair palatogenesis, despite its low baseline expression level (Ozekin et al., 2023). Using the well-established cranial neural crest Cre driven by the *Wnt1* promoter (*Arhgap29^fl/fl^;Wnt1^Cre+^*), we generated neural crest-specific *Arhgap29* knockouts and validated ARHGAP29 knockdown by immunofluorescence. While WT embryos showed strong epithelial and weaker mesenchymal ARHGAP29 staining (Fig. S1C,C′), *Arhgap29^fl/fl^;Wnt1^Cre+^* embryos displayed reduced ARHGAP29 in the palatal mesenchyme compared to WT, while staining was maintained in the oral epithelium and lingual muscle where the driver is not expressed (Fig. S1D,D′). We next assessed whether this loss of ARHGAP29 perturbs embryonic development and evaluated the genotypic frequencies at E14.5, E18.5 and post-weaning (Fig. S1E). Both *Arhgap29^fl/fl^;Wnt1^Cre+^* mutants and littermate controls displayed the expected genotypic frequencies at all three timepoints (Fig. S1E) with no observable macroscopic defects (Fig. S1F-I), indicating that loss of ARHGAP29 within the mesenchyme alone does not affect viability or embryonic development. Lastly, to determine whether this conditional loss of *Arhgap2*9 causes cleft palate, we analyzed coronal sections of E18.5 embryos and found that 100% of both littermate controls and *Arhgap29^fl/fl^;Wnt1^Cre^*^+^ embryos displayed proper palatogenesis (Fig. S1J-O). Together, these data suggest that the mesenchyme-specific loss of *Arhgap29* does not contribute significantly to palatogenesis.

### *Arhgap29-Ect^Cre^*^+^ embryos display gross morphological defects

**Fig. 2.**
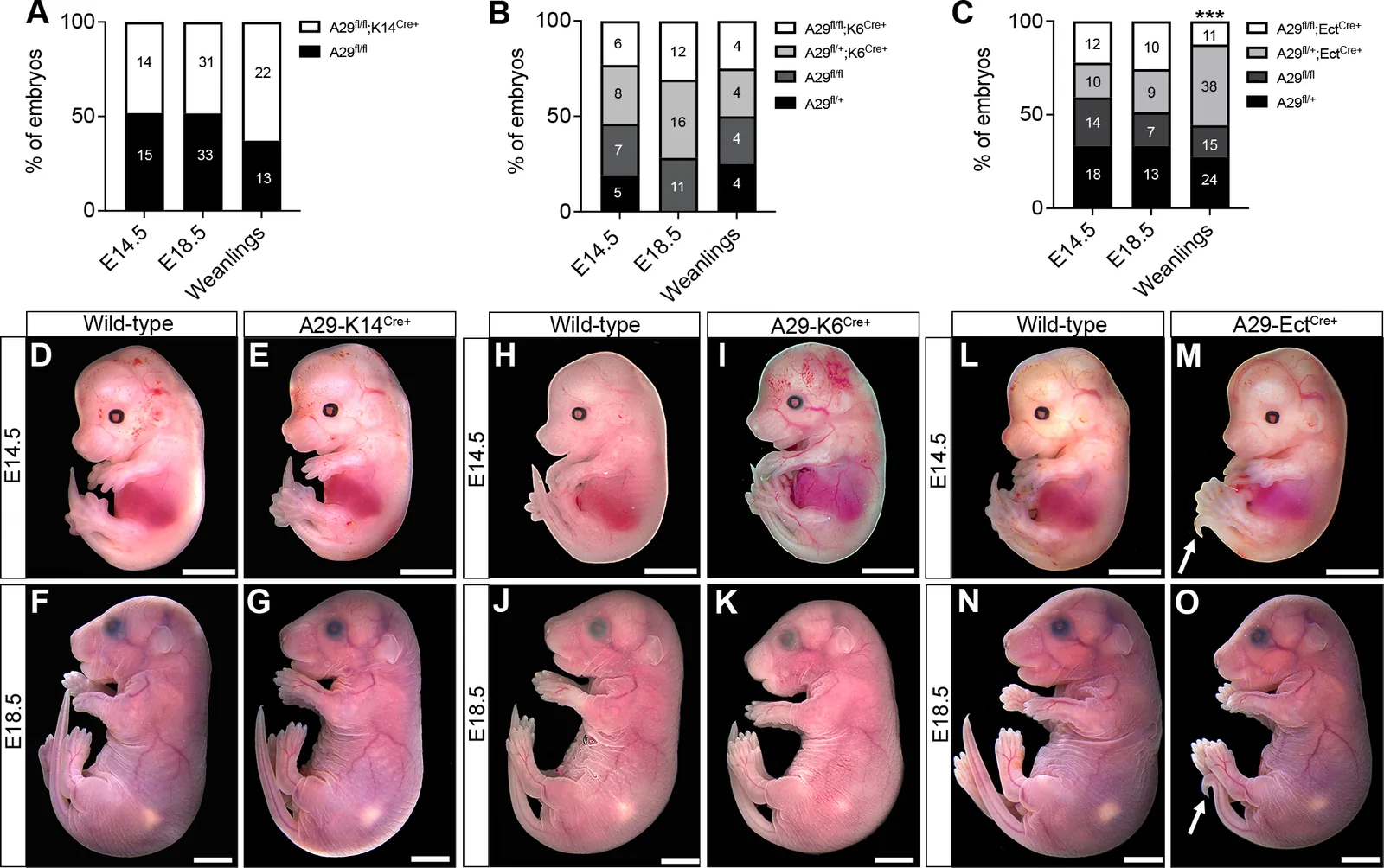
**Loss of ARHGAP29 in all ectoderm-derived cells, but not in basal epithelial cells or periderm only, leads to reduced survival and a kinked tail.** (A-C) Genotypic frequencies expressed as the percentage of embryos (raw numbers shown in each column) at E14.5, E18.5 and weaning. ***P<0.001, Pearson's Chi-squared test. (A) A29-K14^Cre+^, *Arhgap29^fl/fl^;K14^Cre^*^+^; wild-type, *Arhgap29^fl/fl^*. (B) A29-K6^Cre+^, *Arhgap29^fl/fl^;K6^Cre+^*; wild-type, *Arhgap29^fl/fl^* or *Arhgap29^fl/+^*. (C) A29-Ect^Cre+^, *Arhgap29^fl/fl^;Ect^Cre+^*; wild-type, *Arhgap29^fl/fl^* or *Arhgap29^fl/+^*. Note that *Arhgap29^fl/fl^;Ect^Cre+^*, *Arhgap29^fl/+^;K6^Cre^*^+^ and *Arhgap29^fl/+^* were part of the original cross but not further analyzed. (D-O) Macroscopic images of wild-type (D,F,H,J,L,N), *Arhgap29^fl/fl^;K14^Cre+^* (E,G), *Arhgap29^fl/fl^;K6^Cre+^* (I,K) and *Arhgap29^fl/fl^;Ect^Cre+^* (M,O) embryos at E14.5 (D,E,H,I,L,M) and E18.5 (F,G,J,K,N,O). White arrows point to the kinked tail. Scale bars: 250 μm.

**Fig. 3.**
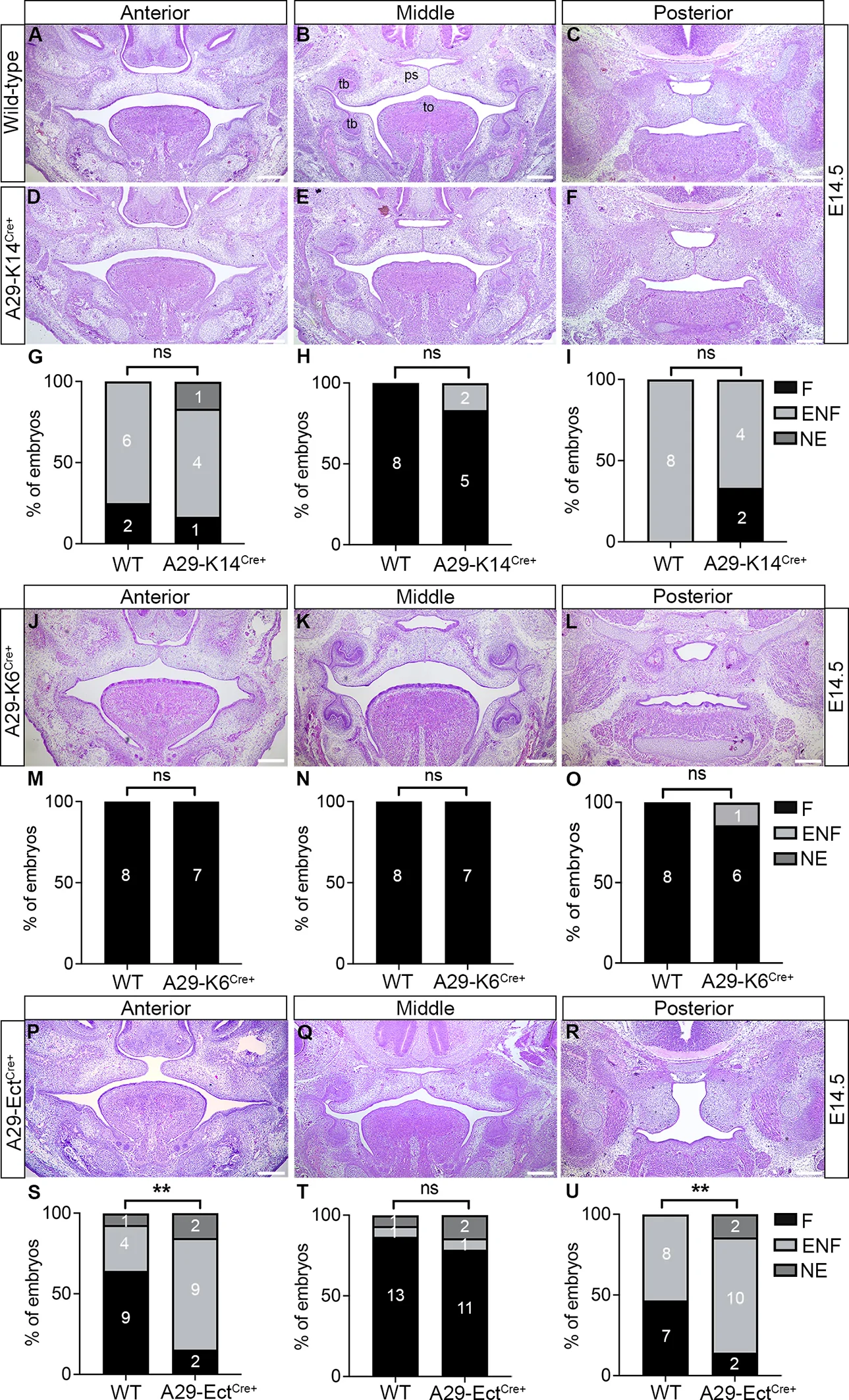
***Arhgap29^fl/fl^;Ect^Cre+^* embryos exhibit delayed palatogenesis at E14.5.** (A-F,J-L,P-R) Coronal sections of E14.5 wild-type (WT) (A-C), *Arhgap29^fl/fl^;K14^Cre+^* (A29-K14^Cre+^; D-F), *Arhgap29^fl/fl^;K6^Cre+^* (A29-K6^Cre+^; J-L) and *Arhgap29^fl/fl^;Ect^Cre+^* (A29-Ect^Cre+^; P-R) embryos, stained with Hematoxylin and Eosin in the anterior (A,D,J,P), middle (B,E,K,Q) and posterior (C,F,L,R) regions of the palatal shelves. (G-I,M-O,S-U) Percentage of embryos (raw numbers shown in each column) with fused (‘F’), elevated but not fused (‘ENF’) or not elevated (‘NE’) palatal shelves. **P<0.01, Fisher's exact test; ns, not significant; ps, palatal shelf; tb, toothbud; to, tongue. Scale bars: 250 μm.

We evaluated the genotypic frequencies of *Arhgap29^fl/fl^;K14^Cre^*^+^, *Arhgap29^fl/fl^;K6^Cre+^* and *Arhgap29^fl/fl^;Ect^Cre+^* embryos at E14.5, E18.5 and post-weaning to assess viability (Fig. 2A-C). *Arhgap29^fl/fl^;K14^Cre^*^+^ and *Arhgap29^fl/fl^;K6^Cre+^* embryos exhibited the expected genotypic frequencies at each of these timepoints (Fig. 2A,B). *Arhgap29^fl/+^;Ect^Cre+^* and *Arhgap29^fl/fl^;Ect^Cre+^* embryos also displayed the expected genotypic frequencies at E14.5 and E18.5 (Fig. 2C). However, the weanling frequency of the *Arhgap29^fl/fl^;Ect^Cre+^* deviated from the expected Mendelian ratio with a significant reduction in the number of mutants compared to littermate controls, suggesting a partial loss of postnatal viability (Fig. 2C). Macroscopically, WT, *Arhgap29^fl/fl^;K14^Cre^*^+^ and *Arhgap29^fl/fl^;K6^Cre+^* embryos were indistinguishable at both E14.5 and E18.5, with no obvious craniofacial abnormalities (Fig. 2D-K). However, *Arhgap29^fl/fl^;Ect^Cre+^* embryos at both E14.5 and E18.5 displayed a fully penetrant kinked tail (Fig. 2L-O), occasional eye defects [5/29 embryos (17%); Fig. S3A-E] and ectrodactyly [2/35 embryos (6%); Fig. S3G,H]. The ocular defect observed in *Arhgap29^fl/fl^;Ect^Cre+^* embryos consists of an atrophic lens potentially resulting from a failure of the optic cup fissure to close, resembling a coloboma (Fig. 3B,D). At weaning, *Arhgap29^fl/fl^;Ect^Cre+^* pups exhibited a significant reduction in body weight that continued through adulthood (Fig. S3F). None of the murine models generated displayed the presence of a cleft lip. Collectively, these results demonstrate that the combined loss of ARHGAP29 in the oral epithelium and periderm reduces viability and results in visible morphological defects, including impaired ocular development.

### ARHGAP29 is required in oral epithelial and periderm cells for proper palatogenesis

To determine the requirement for ARHGAP29 in the oral epithelium and/or the periderm, we performed histological analysis of coronal sections of *Arhgap29^fl/fl^;K14^Cre^*^+^, *Arhgap29^fl/fl^;K6^Cre+^*, *Arhgap29^fl/fl^;Ect^Cre+^* and WT littermate control heads at E14.5. Palatal defects were scored in the anterior, middle and posterior regions of the palatal shelves, as either not elevated, elevated but not fused, or fused. At E14.5, WT (Fig. 3A-C), *Arhgap29^fl/fl^;K14^Cre^*^+^ (Fig. 3D-I) and *Arhgap29^fl/fl^;K6^Cre+^* (Fig. 3J-O) embryos displayed on-time palatogenesis in the three palatal regions. Conversely, *Arhgap29^fl/fl^;Ect^Cre+^* embryos displayed defects that included palatal shelves that were not elevated, or elevated but not fused compared to WT (Fig. 3P-U). Interestingly, these defects were only statistically significant in the anterior and posterior regions of the palate (Fig. 3S-U). These data demonstrate that the combined loss of ARHGAP29 in the oral epithelium and periderm disrupts the timing of palatogenesis and may also suggest a spatial requirement for ARHGAP29 along the antero-posterior axis.

### Loss of ARHGAP29 in ectoderm-derived cells results in cleft palate

**Fig. 4.**
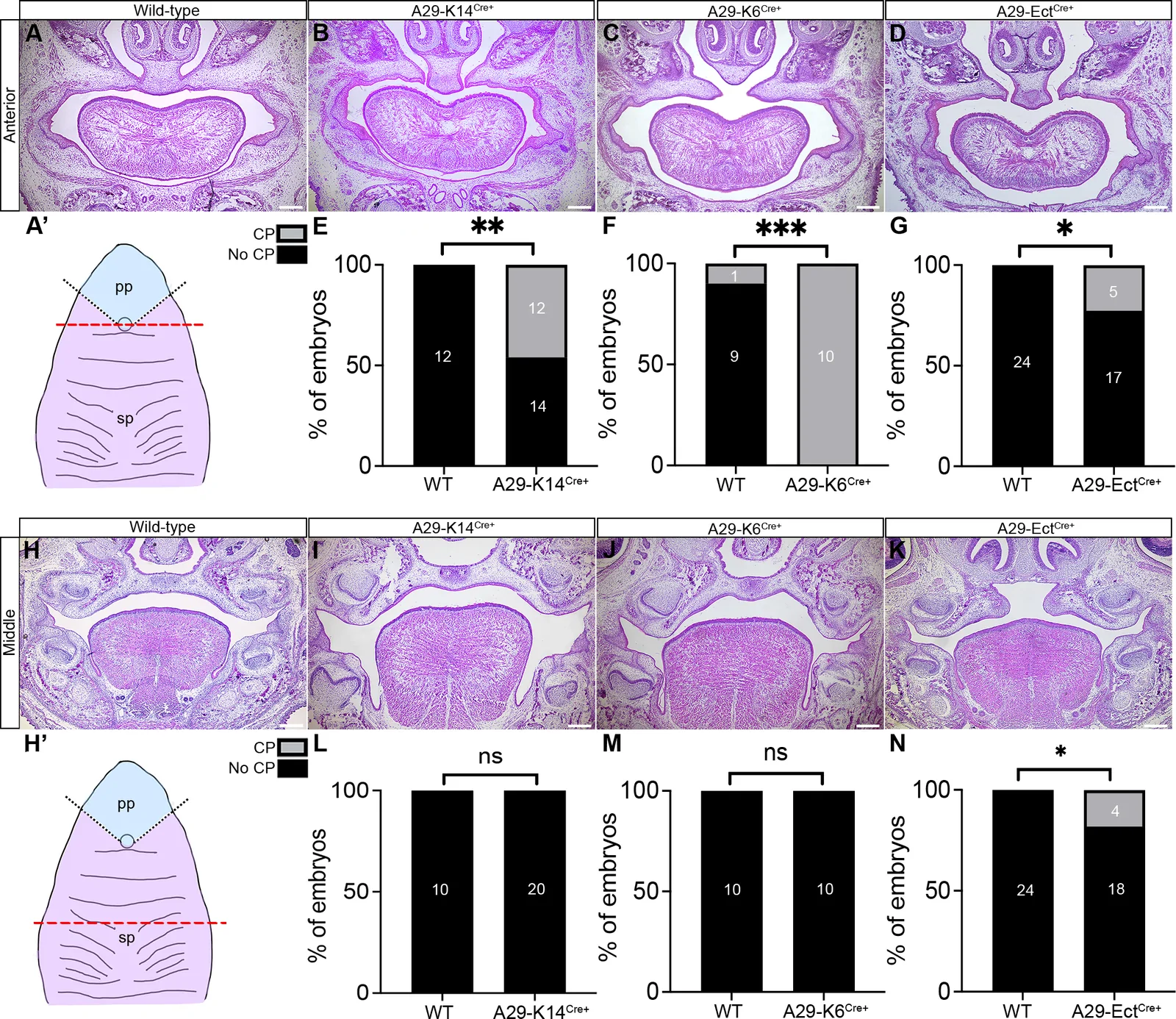
**Loss of ARHGAP29 in all ectoderm-derived cells, alone or in combination, leads to cleft of the secondary palate.** (A-D) Coronal sections of the anterior secondary palate at the intersection between the primary and secondary palate (see red dashed line in A′), from E18.5 wild-type (WT) (A), *Arhgap29^fl/fl^;K14^Cre+^* (A29-K14^Cre+^; B), *Arhgap29^fl/fl^;K6^Cre^*^+^ (A29-K6^Cre+^; C) and *Arhgap29^fl/fl^;Ect^Cre^*^+^ (A29-Ect^Cre+^; D) embryos, stained with Hematoxylin and Eosin. (A′) Schematic showing the roof of the mouth with the primary palate (pp) in light blue and the secondary palate (sp) in lilac. The red dashed line indicates the plane of section. (E-G) Corresponding percentage of embryos (raw numbers shown in each column) with fused palatal shelves (‘No CP’) or cleft palate (‘CP’). (H-M) Coronal sections from the middle of the secondary palate (see red dashed line in H′) from E18.5 wild-type (H), *Arhgap29^fl/fl^;K14^Cre^*^+^ (I), *Arhgap29^fl/fl^;K6^Cre+^* (J) and *Arhgap29^fl/fl^;Ect^Cre^*^+^ (K) embryos, stained with Hematoxylin and Eosin. (H′) Schematic showing the roof of the mouth with the primary palate (pp) in light blue and the secondary palate (sp) in lilac color. The red dashed line indicates the plane of section. (L-N) Corresponding percentage of embryos (raw numbers shown in each column) with fused palatal shelves (‘No CP’) or cleft palate (‘CP’). *P<0.05, **P<0.001, ***P<0.0001, Fisher's exact test; ns, not significant; pp, primary palate; sp, secondary palate. Scale bars: 250 μm.

To determine whether the observed defects in palatogenesis at E14.5 are a simple delay in development or a permanent defect, we scored embryos for the presence of a cleft palate at E18.5. All WT, *Arhgap29^fl/fl^;K14^Cre^*^+^ and *Arhgap29^fl/fl^;K6^Cre+^* embryos displayed a properly formed secondary palate at E18.5 in the middle and posterior regions, whereas 18% of *Arhgap29^fl/fl^;Ect^Cre+^* embryos showed a complete cleft that spanned the full antero-posterior palatal axis (Fig. 4H-N). Interestingly, 46% of *Arhgap29^fl/fl^;K14^Cre^*^+^, 100% of *Arhgap29^fl/fl^;K6^Cre+^* and 22% of *Arhgap29^fl/fl^;Ect^Cre+^* embryos displayed a cleft at the anterior end of the secondary palate where the secondary and primary palates fuse (Fig. 4A-G). This cleft was still present at weaning in 100% of *Arhgap29^fl/fl^;K6^Cre+^* and 40% of *Arhgap29^fl/fl^;K14^Cre^*^+^ mice (Fig. S4). Together, we conclude that ARHGAP29 is required in the ectoderm (oral epithelium and periderm) for palatogenesis.

### ARHGAP29 is required for proper palatal epithelial morphology

**Fig. 5.**
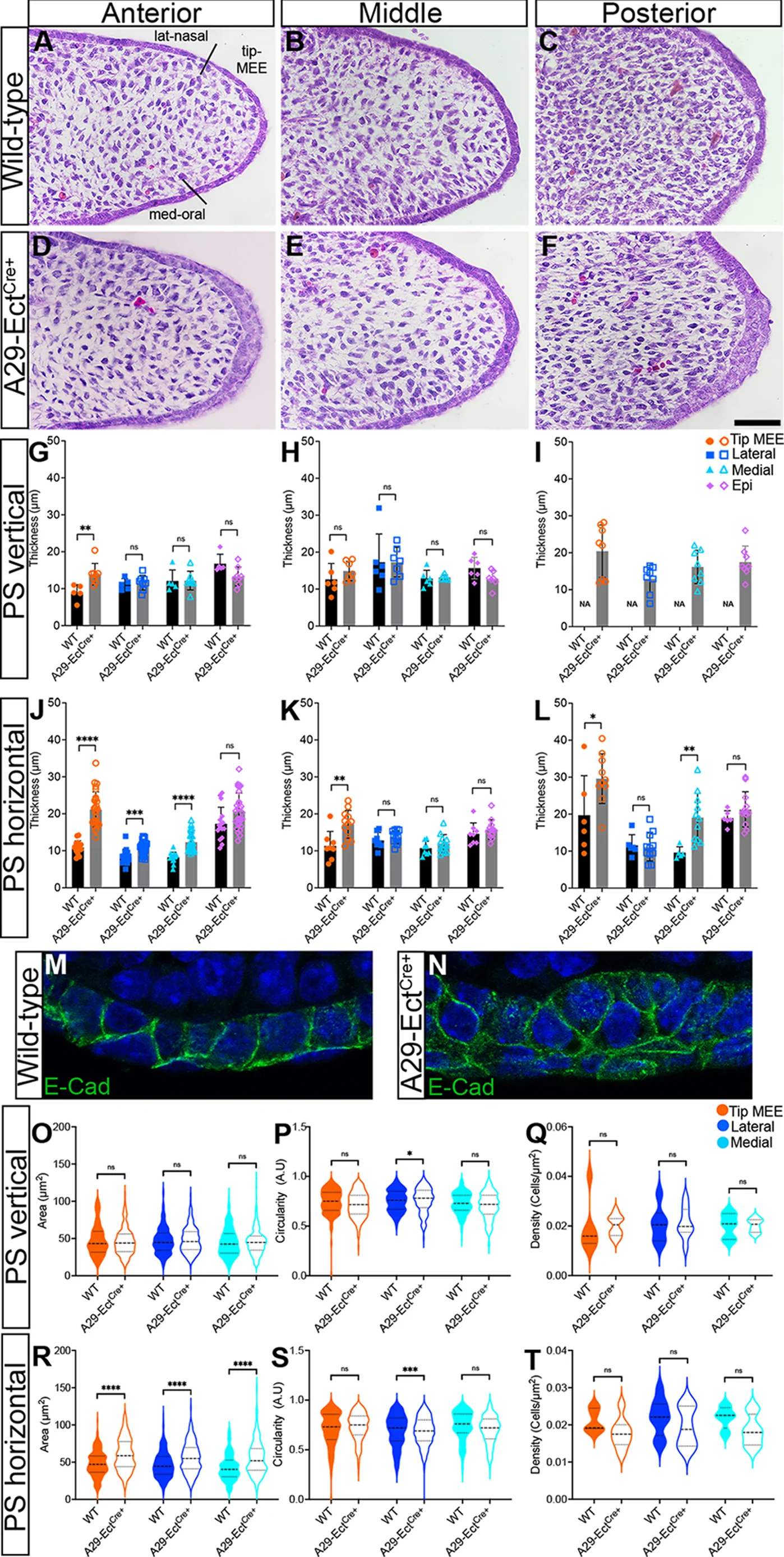
**ARHGAP29 is required for oral epithelial cell morphology.** (A-F) Elevated palatal shelves of E14.5 wild-type (WT) (A-C) and *Arhgap29^fl/fl^;Ect^Cre+^* (A29-Ect^Cre+^; D-F) embryos, stained with Hematoxylin and Eosin at their anterior (A,D), middle (B,E) and posterior (C,F) positions along the antero-posterior axis. Black lines in A separate subregions of the palatal shelf epithelium used for measurements in G-L and O-T (lateral when vertical becomes nasal when horizontal; medial when vertical becomes oral when horizontal; tip of the palatal shelf when vertical becomes MEE when horizontal). Scale bars: 50 μm. (G-L) Quantification of epithelial thickness from WT and *Arhgap29^fl/fl^;Ect^Cre+^* E14.5 palatal shelves in vertical (G-I) and horizontal (J-L) positions as well as thickness of the epidermis below the eye. (M-T) Using E-cadherin staining of WT and *Arhgap29^fl/fl^;Ect^Cre+^* epithelial cells (examples shown in M and N), cell area (O,R), circularity (P,S) and density (Q,T) were quantified. *P<0.05, **P<0.01, ***P<0.001, ****P<0.0001, unpaired Student's *t*-test. NA, not available; ns, not significant; PS, palatal shelves. Error bars represent s.d.

We focused our remaining studies on the complete cleft of the secondary palate (further referred to as cleft palate and palate) phenotype of *Arhgap29^fl/fl^;Ect^Cre+^* embryos as it is incompatible with neonatal murine life (contrary to anterior cleft of the secondary palate; Gu et al., 2008; Sarper et al., 2018; Yu et al., 2005). We noticed a thickening of the epithelium covering the palatal shelves of *Arhgap29^fl/fl^;Ect^Cre+^* embryos, specifically at their distal tip (Fig. 5), the presumptive MEE. To quantify this observation, we measured the thickness of the epithelium along the shelves (three different regions when shelves are down: lateral, tip, medial; when shelves are up: lateral becomes oral, tip becomes MEE, medial becomes nasal) in the anterior, middle and posterior regions of the palate in both embryos with palatal shelves not elevated and embryos with palatal shelves elevated but not fused. Fused shelves have varying degrees of medial edge epithelial cell intercalation, which precludes measurement at this position. *Arhgap29^fl/fl^;Ect^Cre+^* embryos showed a 1.5-2-fold increase in the thickness of the epithelium at the distal tip of the palatal shelves (Fig. 5A-L). This increase was restricted to the anterior region when the shelves were not elevated, and expanded to the entire antero-posterior regions when shelves were elevated (Fig. 5G-L). Additionally, after elevation, the oral and nasal epithelia of *Arhgap29^fl/fl^;Ect^Cre+^* embryos were significantly thicker in the anterior region. Interestingly, as palatogenesis progressed and shelves became elevated, the thickness of the epithelium increased all around the shelves in the anterior region, suggesting a worsening of the phenotype specifically anteriorly. Of note, the thickness of the epidermis around the head was unchanged between experimental groups (Fig. 5G-L) and all WT embryos displayed elevated palatal shelves in the posterior region, precluding the measurement of the epithelial thickness at that palatal position (Fig. 5I). Collectively, these results demonstrate a unique spatial and temporal requirement for ARHGAP29 in controlling epithelial morphology during palatogenesis.

### ARHGAP29 is required for oral epithelial cell morphology but not oral epithelial cell proliferation

Given the thickened palatal epithelium in *Arhgap29^fl/fl^;Ect^Cre+^* mutants, we next investigated epithelial proliferation as a potential cause for this phenotype. We focused on the anterior region of the oral cavity as this is where we observed the most significant change in epithelial thickness. The percentage of marker of proliferation kiel 67 (Ki-67)-positive cells and the percentage of bromodeoxyuridine (BrdU)-positive cells 48 h after injection, were unchanged between WT and *Arhgap29^fl/fl^;Ect^Cre+^* mutants (Fig. S5). These data suggest that the loss of ARHGAP29 does not alter proliferation within the palatal shelf epithelium and mesenchyme at E14.5. We then measured the size of the epithelial cells in this region using E-cadherin as a proxy for the plasma membrane (Fig. 5M,N) and found that when the palatal shelves are unelevated, there is a mild, yet not significant, increase in epithelial cell area, minor changes in circularity, and no change in epithelial cell density, compared to WT (Fig. 5O-Q). Once the palatal shelves were elevated we found a significant increase in cell area in all regions analyzed, a reduction in cellular circularity only on the oral side of the shelves, and no change in cell density (Fig. 5R-T). These results suggest that the ectodermal loss of ARHGAP29 results in a hypertrophic rather than a hyperproliferative palatal epithelium. Interestingly, morphological differences were also observed in the lingual epithelium of *Arhgap29^fl/fl^;Ect^Cre+^* with a loss of classic rosette structures on the dorsum of the tongue, an increase in epithelial cell area and loss of circularity (Fig. S6). Together, these data suggest that ARHGAP29 is not required for oral epithelial proliferation but instead for regulating cell area and shape as the palatal shelves progress through palatogenesis.

### ARHGAP29 suppresses markers of contractility in the MEE

**Fig. 6.**
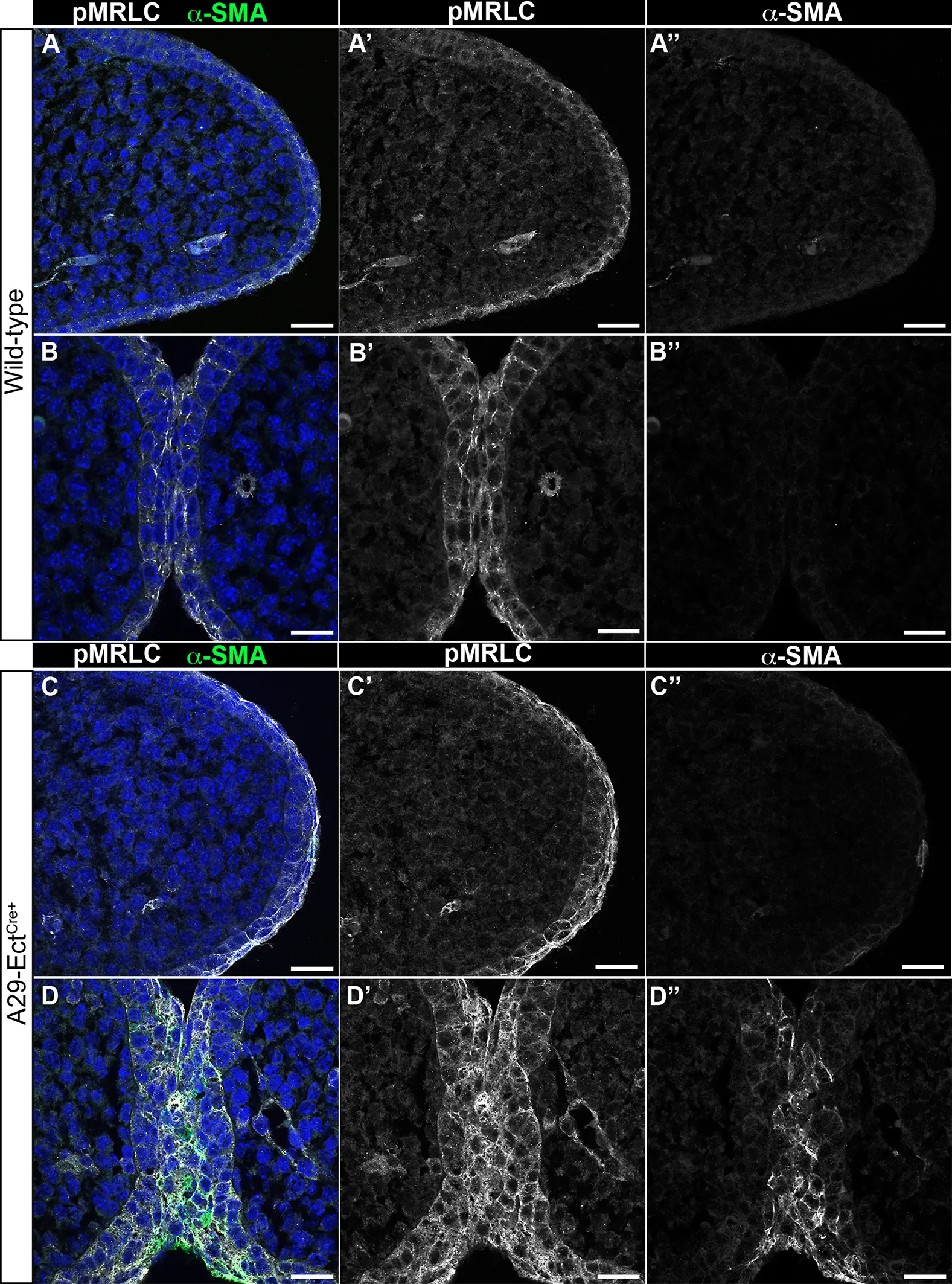
**ARHGAP29 suppresses markers of contractility in the MEE.** (A-D″) Maximum projections of 10-15 confocal slices of elevated (A-A″,C-C″) and fusing (B-B″,D-D″) palatal shelves from E14.5 wild-type (A-B″) and *Arhgap29^fl/fl^;Ect^Cre+^* (A29-Ect^Cre+^; C-D″) embryos dual stained with phospho-myosin regulatory light chain (pMRLC; white in merged) and alpha-smooth muscle actin (α-SMA; green in merged). Scale bars: 20 μm.

Because ARHGAP29 is a regulator of the RhoA pathway, and RhoA is a regulator of cell size, shape and motility (Van Aelst and Symons, 2002), we examined whether the expression or activity of RhoA downstream effectors are affected in *Arhgap29^fl/fl^;Ect^Cre+^* palatal epithelium. We investigated two downstream effectors of RhoA: myosin regulatory light chain (MRLC), which is phosphorylated upon activation of RhoA and promotes contractility of the actin cytoskeleton (Kimura et al., 1996), and α-SMA, which is typically detected in activated myofibroblasts and only present in specialized epithelial cells (Mack et al., 2001; Masszi et al., 2003; Wang et al., 2006). In WT embryos, p-MRLC and α-SMA were absent from unelevated palatal shelves (Fig. S7A-A″). As shelves elevated and fused, p-MRLC was increasingly detected but consistently at lower levels in basal cells compared to superficial cells and was particularly enriched at cell–cell junctions of periderm cells (Fig. 6A,A′,B,B′). α-SMA was absent at all palatal positions in WT (Fig. 6A,A″,B,B″). In *Arhgap29^fl/fl^;Ect^Cre+^* embryos, however, p-MRLC was upregulated throughout the epithelium, particularly in the superficial layers (Fig. 6C,C′,D,D′; Fig. S7B,B′). This increase was observed at all palatal positions and appeared to increase in severity during fusion (Fig. 6D,D′). Concomitantly, levels of α-SMA were increased in *Arhgap29^fl/fl^;Ect^Cre+^* embryos only once the palatal shelves were elevated or were fusing (Fig. 6C,C″,D,D″; Fig. S7B,B″). These results demonstrate that, in the absence of ARHGAP29, contractility markers are upregulated, which may contribute to the morphological changes in the oral and lingual epithelium and could perturb cytoskeletal remodeling required for palatogenesis to occur at the correct developmental time.

### *Arhgap29^fl/fl^;Ect^Cre+^* embryos display defects in epithelial identity but not in epithelial differentiation

**Fig. 7.**
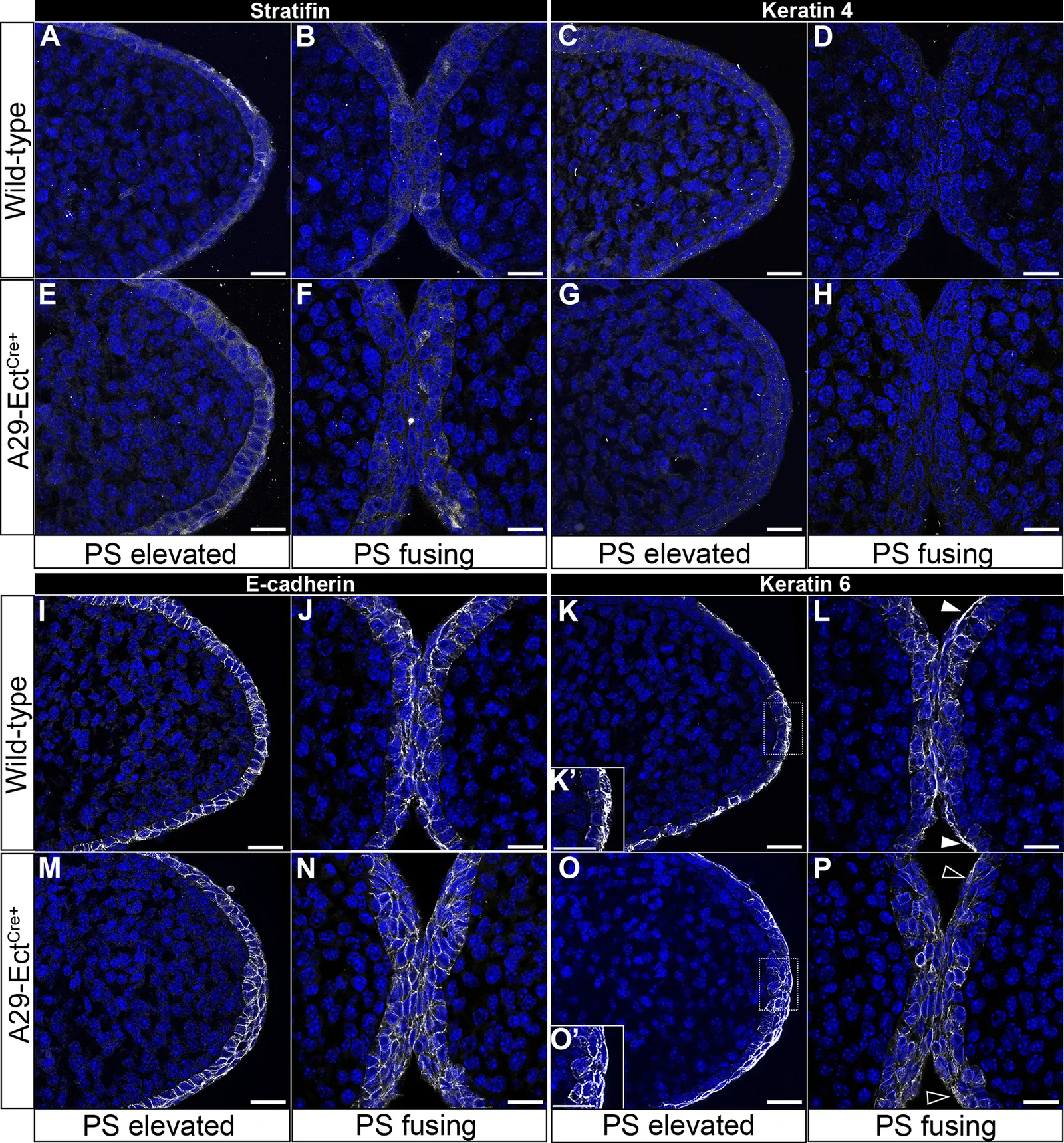
**Periderm and epithelial organization, but not epithelial differentiation, are affected by the loss of ARHGAP29.** (A-P) Maximum projections of two to four confocal slices of elevated and fusing palatal shelves from E14.5 wild-type (A-D,I-L) and *Arhgap29^fl/fl^;Ect^Cre+^* (A29-Ect^Cre+^; E-H,M-P) embryos stained with stratifin (A,B,E,F), keratin 4 (C,D,G,H), E-cadherin (I,J,M,N) or keratin 6 (K,L,O,P). K′ and O′ are higher magnifications of the boxed regions of the same panel, demonstrating changes in epithelial organization in *Arhgap29^fl/fl^;Ect^Cre^*^+^. White solid arrowheads on fusing shelves show periderm cells enriched for keratin 6 in wild type; open arrowheads point to periderm cells that are not enriched for keratin 6 in *Arhgap29^fl/fl^;Ect^Cre+^*. Scale bars: 20 μm.

Epithelial contractility is increased during epidermal differentiation, with the most contractile cells found in the superficial layers (Prado-Mantilla et al., 2025). Therefore, we next investigated whether there is premature or abnormal differentiation occurring in *Arhgap29^fl/fl^;Ect^Cre+^* embryos, by immunostaining for stratifin and keratin 4 (k4) two markers of early oral epithelial differentiation. Stratifin, but not k4, was detected in the MEE and the oral epithelium of WT embryos, at all three palatal positions (Fig. 7A-D; Fig. S8A,B). No differences were observed between WT and *Arhgap29^fl/fl^;Ect^Cre+^* embryos with palatal shelves unelevated, elevated or fused (Fig. 7E-H; Fig. S8C,D), suggesting that premature differentiation is not contributing to the increased contractility markers in these embryos. Next, we investigated the expression of E-cadherin, a cell–cell adhesion protein detected at the plasma membrane of epithelial cells, and k6, a marker of the periderm. In unelevated and elevated WT palatal shelves, the epithelial and periderm cells were each properly organized into a single layer, with E-cadherin detected in all epithelial cells and k6 expression restricted to the periderm (Fig. 7I,K,K′; Fig. S8E,F). During palatal fusion, and consistent with prior reports (Fitchett and Hay, 1989; Teng et al., 2022), k6 expression was expanded into the basal layer of the MEE in WT (Fig. 7L). In *Arhgap29^fl/fl^;Ect^Cre+^* embryos, however, although unelevated palatal shelves displayed proper E-cadherin and k6 distribution (Fig. S8G,H), elevated and fusing palatal shelves exhibited expanded k6 into both basal and periderm cells when palatal shelves were elevated (Fig. 7O,O′) and reduced k6 in the periderm at fusion compared to WT (Fig. 7P), while E-cadherin remained unaffected (Fig. 7M,N). These data demonstrate a loss of specificity in spatial distribution of k6, potentially revealing a lack of lineage specification that might contribute to the changes in epithelial organization.

### *Arhgap29^fl/fl^;Ect^Cre+^* embryos display increased apoptosis during palatal fusion

**Fig. 8.**
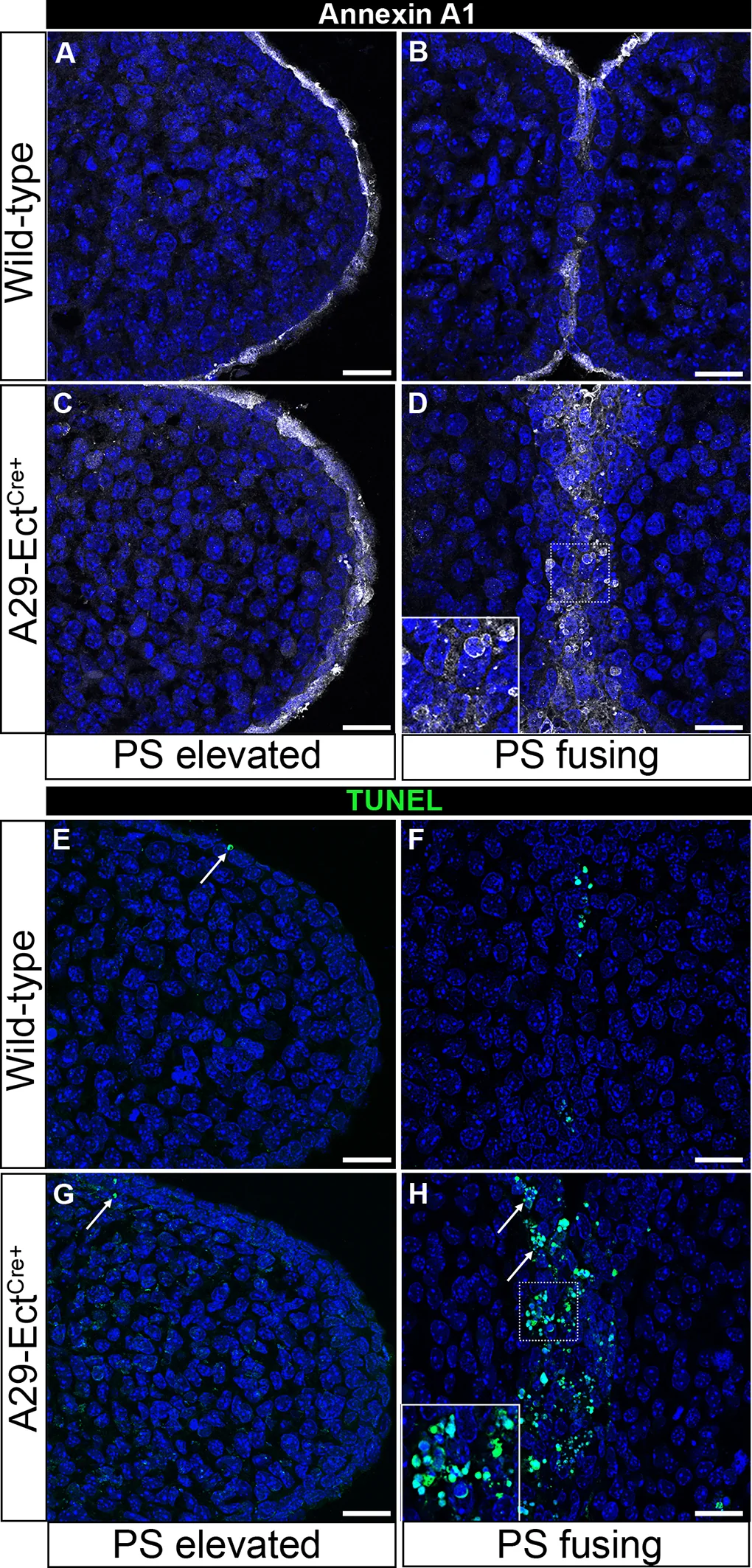
***Arhgap29^fl/fl^;Ect^Cre+^* embryos display increased apoptosis during palatal fusion.** (A-D) Maximum projections of two to four confocal slices of E14.5 wild-type (A,B) and *Arhgap29^fl/fl^;Ect^Cre+^* (A29-Ect^Cre+^; C,D) with elevated (A,C) or fusing (B,D) palatal shelves stained with annexin A1 (gray). (E-H) Coronal sections of E14.5 wild-type (E,F) and *Arhgap29^fl/fl^;Ect^Cre+^* (G,H) with elevated (E,G) and fusing (F,H) palatal shelves stained with TUNEL (green). White arrows point to TUNEL-positive cells. Scale bars: 20 μm.

Apoptosis of MEE cells contribute to palatal fusion; therefore, we evaluated the expression of annexin A1, a protein known to promote apoptosis and that is highly expressed in epithelial and periderm cells (Fankhaenel et al., 2023; Fava et al., 1993). In WT palatal shelves at all three palatal positions, annexin A1 expression was specific to the periderm cells (Fig. 8A,B; Fig. S9A). Although the *Arhgap29^fl/fl^;Ect^Cre+^* palatal shelves also showed the presence of annexin A1 in the periderm cells at all positions, its domain of expression was expanded to multiple superficial layers when the palatal shelves were elevated or fusing (Fig. 8C,D; Fig. S9C). Interestingly, we observed the presence of annexin A1-positive puncta in *Arhgap29^fl/fl^;Ect^Cre+^* fusing palatal shelves that were absent in WT (Fig. 8B,D). These puncta were specific to the MEE, between 1 and 20 μm^2^ in area, and were more numerous in the fused MEE. Given that the size of the annexin A1 puncta was consistent with the size of apoptotic bodies (Battistelli and Falcieri, 2020; Santavanond et al., 2021), we investigated whether these puncta would correlate with apoptosis. TUNEL staining of unelevated and elevated shelves revealed occasional positive cells in both WT and *Arhgap29^fl/fl^;Ect^Cre+^* (Fig. 8E,G; Fig. S9B,D). However, while WT fusing shelves continued to show occasional TUNEL positive cells (Fig. 8F), *Arhgap29^fl/fl^;Ect^Cre+^* fusing shelves had abundant TUNEL-positive cells (Fig. 8H), similar to annexin A1-positive puncta, supporting increased apoptosis in *Arhgap29^fl/fl^;Ect^Cre+^* fusing palatal shelves. This increase in apoptosis may be a compensatory mechanism, enacted upon ARHGAP29 loss, that contributes to the removal of MEE cells and facilitates palatal fusion in the presence of epithelial cells that are more contractile and less motile.

## DISCUSSION

In this study, we used tissue-specific deletion of *Arhgap29* in mice to provide the first direct evidence that ARHGAP29 in ectoderm-derived cells contributes to the proper elevation and fusion of palatal shelves.

We utilized a series of *Arhgap29* conditional knockout murine models to overcome the previously reported embryonic lethality observed in null animals. However, contrary to these earlier findings, a recent publication reported the generation of an *Arhgap29* knockout mouse using CRISPR that survive through gestation (Chi et al., 2025; Zhang et al., 2025). Although these mutants display craniofacial defects, they do not enable the tissue-specific roles of ARHGAP29 to be investigated. Our results show that loss of ARHGAP29 only in the basal epithelium or only in the periderm disrupts the last stages of palatogenesis when the primary and secondary palates fuse. Conversely, deletion of *Arhgap29* in all ectoderm-derived cells results in a significant delay in palatal shelves fusion at E14.5, and a significant yet not fully penetrant cleft palate at E18.5. Because our studies preclude us from following palatogenesis of individual embryos as they develop, we cannot ascertain that an embryo with unelevated palatal shelves at E14.5 would have a cleft palate at E18.5. However, it is tempting to speculate that the 21% of *Arhgap29^fl/fl^;Ect^Cre+^* embryos with palatal shelves not elevated or not fused at E14.5 would be the ones with the most delayed palatogenesis, ultimately leading to the 18% of E18.5 embryos with a cleft palate. Despite only about 20% of embryos exhibiting a cleft palate, this incomplete penetrance has occasionally been observed in murine models in which genes causing human clefting are deleted, such as *Afdn*, *Specc1l* or *Grhl3* (Awotoye et al., 2024; Hall et al., 2020; Lough et al., 2020; Peyrard-Janvid et al., 2014).

**Fig. 9.**
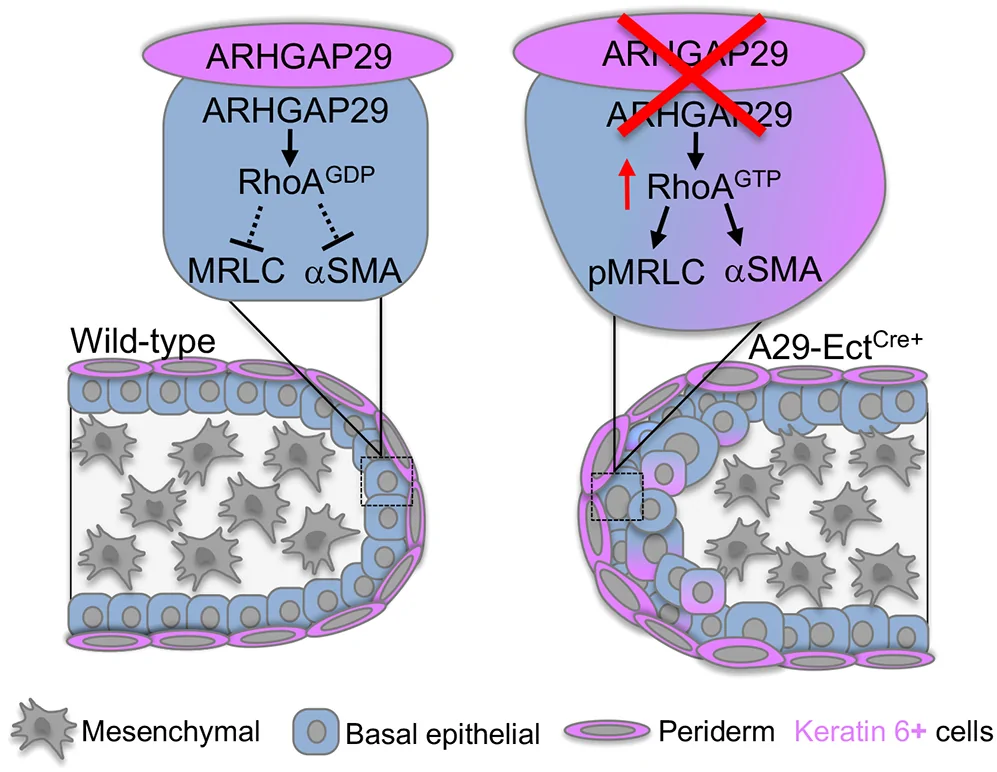
**Proposed model for the role of ARHGAP29 during palatogenesis.** Left: ARHGAP29 within the basal epithelium and periderm of the wild-type palatal shelf functions to inactivate RhoA, which limits the expression of phosphorylated myosin regulatory light chain (pMRLC) and alpha-smooth muscle actin (α-SMA). Right: Loss of ARHGAP29 in the basal epithelium and periderm of *Arhgap29^fl/fl^;Ect^Cre+^* (A29-Ect^Cre+^) embryos results in increased epithelial thickness, increased epithelial cell size, and defects in epithelial organization. Loss of ARHGAP29 results in the increase of pMRLC and α-SMA. Collectively, these defects in epithelial organization, morphology and contractility may perturb the dynamic remodeling of the palatal shelves required for their elevation and fusion.

ARHGAP29 belongs to the RhoA pathway, which has been previously implicated in OFC (Biggs et al., 2014; Ibrahim and Richman, 2026; Leslie et al., 2012), yet how it may contribute to palatogenesis has been unclear because of a lack of useful tools, including our previous *Arhgap29* loss-of-function allele due to early lethality (Paul et al., 2017). Immediately downstream of ARHGAP29 and RhoA is the ROCK/myosin signaling pathway. Activation of this pathway results in increased filamentous actin and phosphorylation of MRLC. Dynamic changes in the actomyosin cytoskeleton are known to be required for proper palatogenesis (Fitchett and Hay, 1989; Goering et al., 2025; Kim et al., 2015; Lessard et al., 1974; Martinez-Alvarez et al., 2000; Teng et al., 2022). Additionally, murine mutants for β-catenin (*Ctnnb1*), *Specc1l* and nectin genes (*Nectin1* and *Nectin4*), modulators of the actomyosin cytoskeleton, display cleft palate. Consistent with these studies, we found that loss of *Arhgap29* early during facial development leads to increased levels of phosphorylated MRLC in oral epithelial cells, suggesting that altered actomyosin dynamics contributes to the mechanism by which ARHGAP29 promotes palatogenesis (Fig. 9). These observations are further supported by our previous *in vitro* studies showing that cutaneous keratinocytes knocked down for *ARHGAP29* display increased filamentous actin and phosphorylated MRLC compared to control (Rhea et al., 2025). Along those lines, we also detect the presence of α-SMA in epithelial cells at the tip of the palatal shelves in *Arhgap29^fl/fl^;Ect^Cre+^* embryos. α-SMA is expressed mostly in fibroblasts when they become contractile but is also detected in some epithelial cells with contractile properties such as in the submucosal glands of the airway (Ding et al., 2021; Korol et al., 2016; Mack et al., 2001; O'Connor et al., 2016). Outside of one report detailing the presence of α-SMA in the oral epithelium of fusing palatal shelves in rat (Gibbins et al., 1999), there is currently no data to suggest a role for this protein in normal palatogenesis. We believe that the presence of α-SMA in conjunction with an increase in phosphorylated MLRC could increase the overall contractility of epithelial cells of the palatal shelves, rendering them stiffer and less amenable to remodeling during palatogenesis (Fig. 9). Functional testing of the contractile properties of the palatal epithelium would be required to demonstrate such a mechanism.

In order for palatogenesis to complete, shelves need to fuse and MEE cells are removed. Migration, dedifferentiation and apoptosis of these cells have all been previously proposed as contributing mechanisms (Bush and Jiang, 2012; Lan et al., 2015). Although apoptosis occurs during palatal shelf fusion, it is not required, consistent with findings reported during fusion of the upper lip (Teng et al., 2025, 2022). Surprisingly, we observed abundant apoptosis in the fusing epithelia of *Arhgap29^fl/fl^*;*Ect^Cre+^* embryos. Along with the presence of apoptotic cells, we observe concomitant expression of annexin A1-positive puncta throughout the MEE. While there is no known role for annexin A1 during palatogenesis, it has well-described roles in mediating apoptosis, primarily in the context of immunity and cancer (Sheikh and Solito, 2018; Sugimoto et al., 2016; Yap et al., 2020). Our data suggest that, in the absence of ARHGAP29, annexin A1 may promote apoptosis as a compensatory mechanism to remove the MEE and facilitate efficient palatal shelf fusion in the presence of MEE cells that are more contractile and potentially less migratory.

Our detailed observations reveal epithelial hypertrophy in *Arhgap29^fl/fl^;Ect^Cre+^* mutants, particularly at the tip of the palatal shelves. This phenotype is reminiscent of the oral epithelial thickening observed in the *Tbx1* knockout mouse (Funato et al., 2012). However, contrary to what we observe in our mutants, the thickening in the *Tbx1* knockout embryos results from abnormal epithelial differentiation and a decrease in proliferation. Epithelial hypertrophy has also been reported in palatal organ cultures treated with nocodazole, a drug that disrupts microtubules (Kitase and Shuler, 2012). Interestingly, this defect does not implicate cell proliferation, but involves altered cytoplasmic localization of ARHGEF2, a microtubule-associated Rho GEF that couples microtubule dynamics to cell contractility. Interplay between the RhoA-ROCK-actomyosin pathway and microtubules is well established (Guan et al., 2023) and microtubule dynamics have been shown previously to be involved in palatogenesis (Hall et al., 2020; Kitase and Shuler, 2013), although there is currently no evidence of a role for ARHGAP29 in regulating microtubule dynamics. Epithelial thickening without changes in proliferation is also evident in the epidermis of mice conditionally lacking laminin 332 in keratinocytes (Tayem et al., 2021). In these mice, α-SMA is strongly upregulated in the superficial epidermal layers, and the shape of epidermal cells is altered. This is consistent with our findings, where loss of ARHGAP29 leads to larger epithelial cells around the palatal shelves with reduced oral epithelial circularity. This increase in size likely accounts for the hypertrophic epithelium observed in these mutants, and is consistent with our previous findings *in vitro* showing a significant increase in cell area and altered morphology in *ARHGAP29* knockdown keratinocytes (Rhea et al., 2025). Cell–cell and cell–matrix adhesions often influence cell shape and size, and ARHGAP29 is involved in endothelial cell adhesion dynamics during tubulogenesis (Barry et al., 2016). Whether ARHGAP29 contributes to similar adhesion remodeling during palatal shelf elevation is currently unknown.

We previously reported a loss-of-function *Arghap29* allele and showed the presence of oral tissue–tissue adhesions (e.g. between oral and lingual epithelia) in heterozygous embryos (Paul et al., 2017). We originally postulated that this phenotype was due to the reduction of ARHGAP29 in the periderm, and that despite periderm cells being present in these embryos, somehow the reduction of ARHGAP29 was impairing periderm function. To our surprise, we did not observe oral adhesions in *Arhgap29^fl/fl^;K14^Cre^*^+^, *Arhgap29^fl/fl^;K6^Cre+^* or *Arhgap29^fl/fl^;Ect^Cre+^* (Fig. 3). The lack of oral adhesions in the *Arhgap29^fl/fl^;K14^Cre^*^+^ can likely be explained by the fact that ARHGAP29 is still present in the periderm. However, their absence in *Arhgap29^fl/fl^;K6^Cre+^* and *Arhgap29^fl/fl^;Ect^Cre+^* is more puzzling. Because the loss-of-function heterozygous model led to a 50% reduction of ARHGAP29 from time of conception in all cell types, and the changes in ARHGAP29 levels in *Arhgap29^fl/fl^;Ect^Cre+^* mutants occurred mid-gestation in ectoderm, one may speculate that there is a temporal or non-ectodermal requirement for ARHGAP29 in preventing oral adhesions. Additionally, our previous loss-of-function heterozygous model was in a full C57BL6/J background, whereas the murine models described in the current study have mixed genetic backgrounds, suggesting that the more complex genetic background of the *Ect^Cre^* and *K6^Cre^* drivers may also contribute to this divergent phenotype.

Although palatal shelves appear as homogeneous outgrowths from the maxillary processes in the oral cavity, molecular heterogeneity along the antero-posterior axis is well established (Bush and Jiang, 2012). Our results extend this heterogeneity to cellular morphology as the oral epithelial hypertrophy in *Arhgap29^fl/fl^;Ect^Cre+^* mutants is initially only detected at the tip of the palatal shelves when they are vertical, and then expands to the medial and lateral epithelia covering the shelves following their elevation. Even more striking is that the defect is restricted to the anterior region of the palate when the shelves are vertical and then extends to the posterior region after their elevation. How palatal shelves change from a vertical to a horizontal position is still unclear; Coleman (1965) and Walker (Walker and Fraser, 1956) previously proposed a heterogeneous mechanism along the antero-posterior axis, whereby the anterior part of the palatal shelves elevate by a ‘flip-up’ process, while the posterior and middle parts of the palatal shelves elevate by a remodeling mechanism (Chiquet et al., 2016; Coleman, 1965; Walker and Fraser, 1956). It is also clear that physical forces are involved (Goering et al., 2025), yet how they contribute to the changes in palatal shelf positions is not well understood. Because ARHGAP29 regulates the actomyosin cytoskeleton, it is tempting to speculate that it may contribute to force generation required for changing palatal shelf position, and may do so heterogeneously along the antero-posterior axis, further supporting different mechanisms for palatal shelf elevation.

Given our previous studies on ARHGAP29 and single-cell RNA sequencing datasets of embryonic facial structures, we know that ARHGAP29 is predominantly detected in ectoderm-derived cells (Joost et al., 2018; Leslie et al., 2012; Paul et al., 2017; Rhea et al., 2025). This ectoderm enrichment is why we focused on Cre drivers that would target epithelial cells. However, given that ARHGAP29 is also detected at a low level in the mesenchyme, we still investigated its possible contribution to palatogenesis and show ARHGAP29 in the mesenchyme is not required for palatogenesis. Our results comparing the *Arhgap29^fl/fl^;K14^Cre^*^+^, *Arhgap29^fl/fl^;K6^Cre+^* and the *Arhgap29^fl/fl^;Ect^Cre+^* models also demonstrate that ARHGAP29 in basal oral epithelial cells alone or periderm alone is not required for fusion between palatal shelves. Its removal from all ectoderm-derived cells (oral basal and periderm), however, impairs palatogenesis and disrupts other developmental processes (e.g. kinked tail, eye defect, low body weight). Despite partial recombination outside of ectoderm-derived cells using this driver, our data from the *Arhgap29^fl/fl^;Wnt1^Cre+^* model confirm that the palatal defects are due solely to the loss of ARHGAP29 in ectoderm-derived cells. Curiously, we observed a higher penetrance of cleft between the primary and secondary palates in *Arhgap29^fl/fl^;K14^Cre^*^+^ and *Arhgap29^fl/fl^;K6^Cre+^* compared to the *Arhgap29^fl/fl^;Ect^Cre+^* mutants, which displayed complete cleft palate at a lower penetrance than the other two models. Since the fusion between the primary and secondary palate is the last fusion to occur, perhaps the basal-only and periderm-only loss of ARHGAP29 results in a less severe delay in palatogenesis, or it could support an increased role for ARHGAP29 in the anterior region of the palate. Additionally, the variation in penetrance could be influenced by a putative distinct role for ARHGAP29 in oral epithelial versus periderm cells, slight differences in the onset or recombination efficiency of the Cre drivers, or the different genetic strain backgrounds during palatogenesis. *Arhgap29^fl/fl^;K14^Cre^*^+^ embryos display a ∼50% penetrant anterior cleft palate compared to the 100% penetrance of *Arhgap29^fl/fl^;K6^Cre+^*, which could be due to the fact that the *K14^Cre^* driver comes on at a slightly later embryonic developmental timepoint than the *K6^Cre^*. However, if this is true we would hypothesize that the *Arhgap29^fl/fl^;Ect^Cre+^* mutants would have a more severe delay or at least an equally penetrant cleft between primary and secondary palates. *Arhgap29^fl/fl^;Ect^Cre+^* mutants display epithelial hypertrophy that the *Arhgap29^fl/fl^;K14^Cre^*^+^ and *Arhgap29^fl/fl^;K6^Cre+^* mutants do not, which is particularly severe in the anterior region of the palate. It is tempting to speculate that this thickening may be enough to bridge the gap between the primary and secondary palates, allowing the epithelia to make sufficient contact for fusion; however, this would need to be formally assessed.

In conclusion, by properly organizing epithelial cells around the palatal shelves and modulating the expression of contractility proteins, ARHGAP29 may participate in the morphological remodeling of the palatal shelves and play a crucial role in time-sensitive embryonic tissue dynamics. Learning how ARHGAP29 integrates with other signaling pathways will further our understanding of the movement of palatal shelves during normal development and OFCs.

## MATERIALS AND METHODS

### Generation and processing of experimental mouse lines

All animal procedures were approved by the Animal Care and Use Committee of the University of Iowa. All mice utilized in this study, except *Ect^Cre^* and *K6^Cre^*, were of C57BL/6J background. Female mice homozygous for the *Arhgap29* floxed (*Arhgap29^fl/fl^*) allele (Barry et al., 2016) were crossed with *Arhgap29^fl/fl^;Wnt1^Cre+^* [129S4.Cg-*E2f1>Tg(Wnt1-cre)2Sor*/J] males (Lewis et al., 2013). Female mice homozygous for both the 3xFLAG-tagged *Arhgap29^fl/fl^* [generated at the University of Iowa Core Facility using an *Arhgap29^fl^* allele (Barry et al., 2016) to CRISPR knock-in 3xFLAG to the second exon immediately following the translation start site] and for the R26-stop-EYFP (*ROSA^yfp/yfp^*; The Jackson Laboratory, strain #006148) were bred with *Arhgap29^fl/fl^*;*ROSA^yfp/yfp^*;*Cre^+^* males, harboring either *K14^Cre^* [*Tg(Krt14-cre)1Efu*], *K6^Cre^* [*Krt6a^em1(icre)Vk^*] or *Ect^Cre^* [*Tg(Tcfap2a-cre)1Will*]. *Wnt1^Cre^* is a transgene driven by the mouse *Wnt1* promoter and enhancer and is active at E8.5 in cranial neural crest (Lewis et al., 2013). *K14^Cre^* is a transgene driven by the human *KRT14* promoter and is fully activated by E11.75 in basal epithelial cells (Vasioukhin et al., 1999). *K6^Cre^* drives *iCre* expression using the endogenous *Krt6* promoter and is active at E11.5 (Saroya et al., 2023). *Ect^Cre^* is driven by an upstream ectodermal enhancer element from *Tcfap2a* and is active at E8.5 in all ectoderm-derived cells (Schock et al., 2017). The presence of a copulatory plug was designated as E0.5. Females were injected with BrdU (5 mg/g body weight) intraperitoneally 2 h prior to euthanasia then euthanized with CO_2_. Litters were harvested at E14.5 and E18.5 as previously described (Paul et al., 2017). Heads were dissected and fixed in 4% paraformaldehyde (PFA) and tail snips or yolk sac used for genotyping. Heads were further processed and embedded in paraffin.

### Histological analysis and phenotype scoring

Serial coronal paraffin sections (7 μm thick) were obtained and stained with Hematoxylin and Eosin for histological analysis. Images were acquired using a Nikon Eclipse E-600 and measurement of palatal epithelium thickness was performed using Nikon NIS-Element software. Although embryos were harvested 14 days after male and female were put together in a cage, serial sectioning revealed phenotypic variations amongst litters as judged by standardized criteria of E14.5 craniofacial development (palatal shelves elevated and opposite epithelia in contact). To provide consistency in our analyses, we applied the same filtering criteria we previously developed (Paul et al., 2017). Briefly, we only considered the following litters for our analysis: (1) they contained at least one WT to be used as internal reference and (2) at least one of these WT demonstrated elevated and fused palatal shelves. Using these criteria, we analyzed 16 litters of *Arhgap29^fl/fl^;K14^Cre+^*, four litters of *Arhgap29^fl/fl^;K6^Cre+^* and nine litters of *Arhgap29^fl/fl^;Ect^Cre+^* at E14.5. This filtering strategy was not necessary at later timepoints. Palatal shelf positions were scored as ‘not elevated’ (vertical on the side of the tongue), ‘elevated but not fused’ (horizontal above the tongue with a gap in the midline between them) and ‘fused’ (shelves in contact with MEE or medial edge seam still present). Palatal regions were defined as anterior (immediately anterior to toothbuds), middle (eye and toothbuds in the field) and posterior (presence of medial pterygoid processes).

### Fluorescence immunostaining and microscopy

Immunostaining of paraffin sections was performed as previously described (Broussard et al., 2021). Sections were de-paraffinized, permeabilized with 0.5% Triton X-100 in PBS and then treated with 0.05% Tween-20 citrate buffer antigen retrieval. Samples were blocked with 2% normal goat serum (Vector Laboratories) in 1% bovine serum albumin in PBS overnight at 4°C, stained in primary antibody at 37°C for 2 h and stained with secondary antibody for 1 h at 37°C. Nuclear DNA was labeled with Hoechst 33342 (1/10,000). Samples were mounted using ProLong™ Diamond Antifade Mountant. Images were acquired with a Zeiss 980 confocal microscope and processed using Fiji software (Schindelin et al., 2012).

### Proliferation analysis by BrdU and Ki-67 detection

BrdU and Ki-67 were detected following immunofluorescence staining as previous described (Paul et al., 2017). Tiled confocal images of the oral cavity were acquired and processed using Fiji software. The percentage of proliferating cells was calculated by dividing the number of BrdU^+^ or Ki-67^+^ cells by the total number of cells as identified by positive nuclear Hoechst staining. Palatal shelves were defined by drawing a vertical line from the palatal hinge region on the nasal side to the oral side of the shelf. Cells were counted in the palatal shelf epithelium and mesenchyme.

### Apoptosis analysis using TUNEL

The DeadEnd™ fluorimetric TUNEL system (Promega) was used to label double-stranded DNA breaks. Paraffin-embedded slides (as described above) were de-paraffinized, washed with 0.85% NaCl, followed by a PBS wash. Slides were fixed with 4% PFA for 15 min at room temperature then washed with PBS. For a positive control, tissue sections were treated with DNase I followed by PBS washes. Sections were incubated in 100 ml equilibration buffer for 10 min followed by incubation in 25 ml of Tdt incubation buffer for 1 h at 37°C, in the dark. Reaction was terminated by submerging slides in a 1:10 solution of 20× SSC in deionized water for 15 min at room temperature, followed by PBS washes. Nuclei were labeled with Hoechst (1/10,000). Samples were mounted using ProLong^TM^ Diamond Antifade Mountant. Images were acquired with a Zeiss 980 confocal microscope and processed using Fiji software.

### Epithelial cell morphology analysis

Immunofluorescence staining was performed on E14.5 coronal sections as described above. Embryos with elevated but not fused palatal shelves were stained for E-cadherin and Hoechst 33342 to label the cell membrane and nuclear DNA, respectively. Confocal images of the oral cavity were acquired and processed using Fiji software. Individual cells were manually traced using the E-cadherin staining, and cell area and circularity measured using Fiji software. The thickness of the epithelium was measured in three different regions along the shelves (when shelves were down: lateral, tip, medial; when shelves were up: lateral becomes oral, tip becomes MEE, medial becomes nasal) in the anterior, middle and posterior regions of the palate in both embryos with palatal shelves not elevated and embryos with palatal shelves elevated but not fused. Fused shelves had varying degrees of medial edge epithelial cell intercalation, which precluded measurements at this position.

### Antibodies

A list of all antibodies used in the study is provided in Table S1.

## Supplementary Material



10.1242/develop.205636_sup1 Supplementary information
