# Supplementary material for: Ectodermal loss of ARHGAP29 alters epithelial morphology and disrupts murine palatogenesis
Source: Development. 2026 Jun 29;153(12):dev205636. doi: 10.1242/dev.205636 (PMC13594415; doi:10.1242/dev.205636)
Supplement: Supplementary file 1 [file develop-153-205636-s1.pdf]

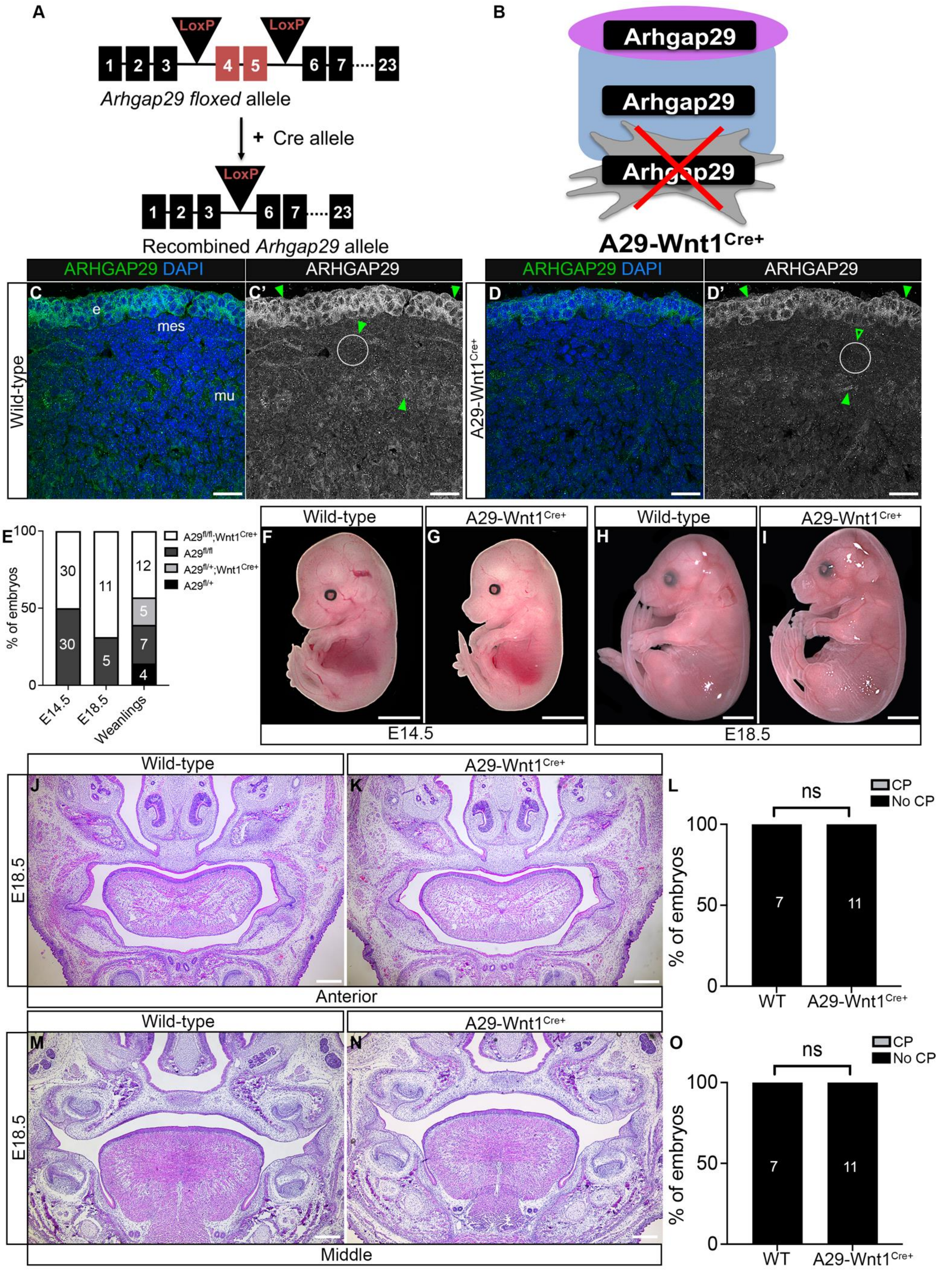

Development • Supplementary information

Fig. S1. ARHGAP29 is not required in the mesenchyme for proper palatogenesis

(A) *Arhgap29 floxed* allele schematic. (B) Schematic of Wnt1^{Cre}-mediated tissue specific deletion of *Arhgap29*. (C-D) Maximum projections of full Z-stacks from ARHGAP29 immunostaining of wild-type (C-C') or *Arhgap29*$^{fl/fl}$;*Wnt1*$^{Cre+}$ (A29-Wnt1^{Cre+}, D-D'). Green channel shown in black and white in C' and D'. Open or full green arrowheads indicate cells (or tissue area) lacking or expressing ARHGAP29 respectively. (E) Genotypic frequencies expressed as percentage of embryos (raw numbers shown in each column) at E14.5, E18.5, and weaning. (F-I) Macroscopic images of wild-type (F,H) and *Arhgap29*$^{fl/fl}$;*Wnt1*$^{Cre+}$ (G,I) embryos at E14.5 (F,G) and E18.5 (H,I). (J,K) Coronal sections of the anterior palate, at the intersection where the primary and secondary palates fuse, from E18.5 wild-type (J) and *Arhgap29*$^{fl/fl}$;*Wnt1*$^{Cre+}$ (K) embryos, stained with Hematoxylin and Eosin. (L) Corresponding percentage of embryos (raw numbers shown in each column) without cleft palate ('No CP') or with cleft palate ('CP'). (M,N) Coronal sections of the middle of the palate at E18.5 from E18.5 wild-type (M), and *Arhgap29*$^{fl/fl}$;*Wnt1*$^{Cre+}$ (N) embryos, stained with Hematoxylin and Eosin. (O) Corresponding percentage of embryos (raw numbers shown in each column) with or without cleft palate. *$P < 0.05$, **$P < 0.001$, ***$P < 0.0001$, Fisher's exact test; ns = not significant. Scale bars for macroscopic images and histology: 250 µm. Scale bars for immunofluorescent staining: 25 µm. e, epithelium; mes, mesenchyme; mu, muscle.

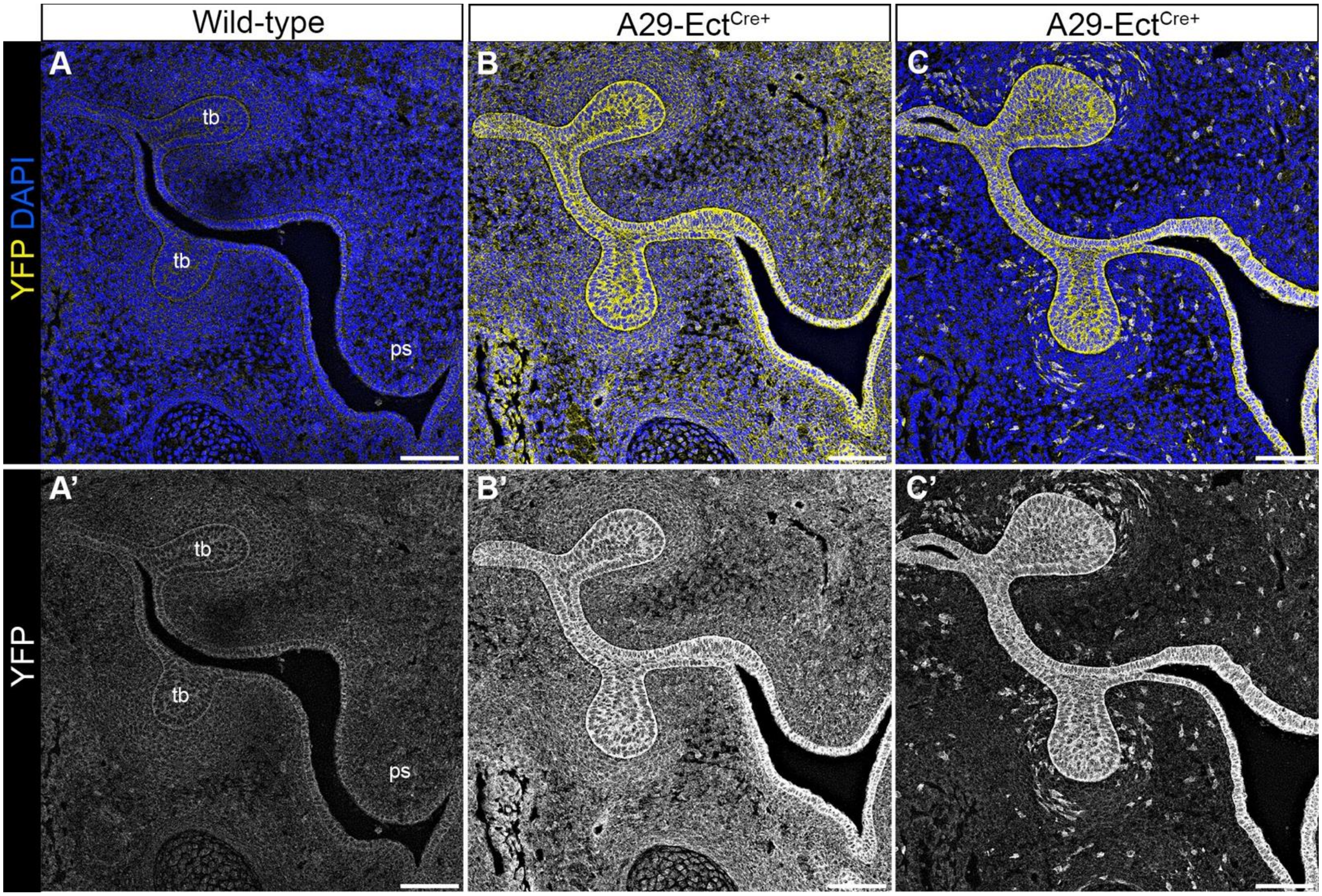

Fig. S2. Characterization of Ect^{Cre} expression pattern. (A-C') Confocal images of the oral cavity (coronal sections) of E14.5 wild-type (A,A') and *Arhgap29$^{fl/fl}$;Ect^{Cre+}* (A29-Ect^{Cre+}, B-C') embryos, stained with YFP (yellow in merged). ps, palatal shelf; tb, tooth bud. Scale bars: 25 μm.

Development • Supplementary information

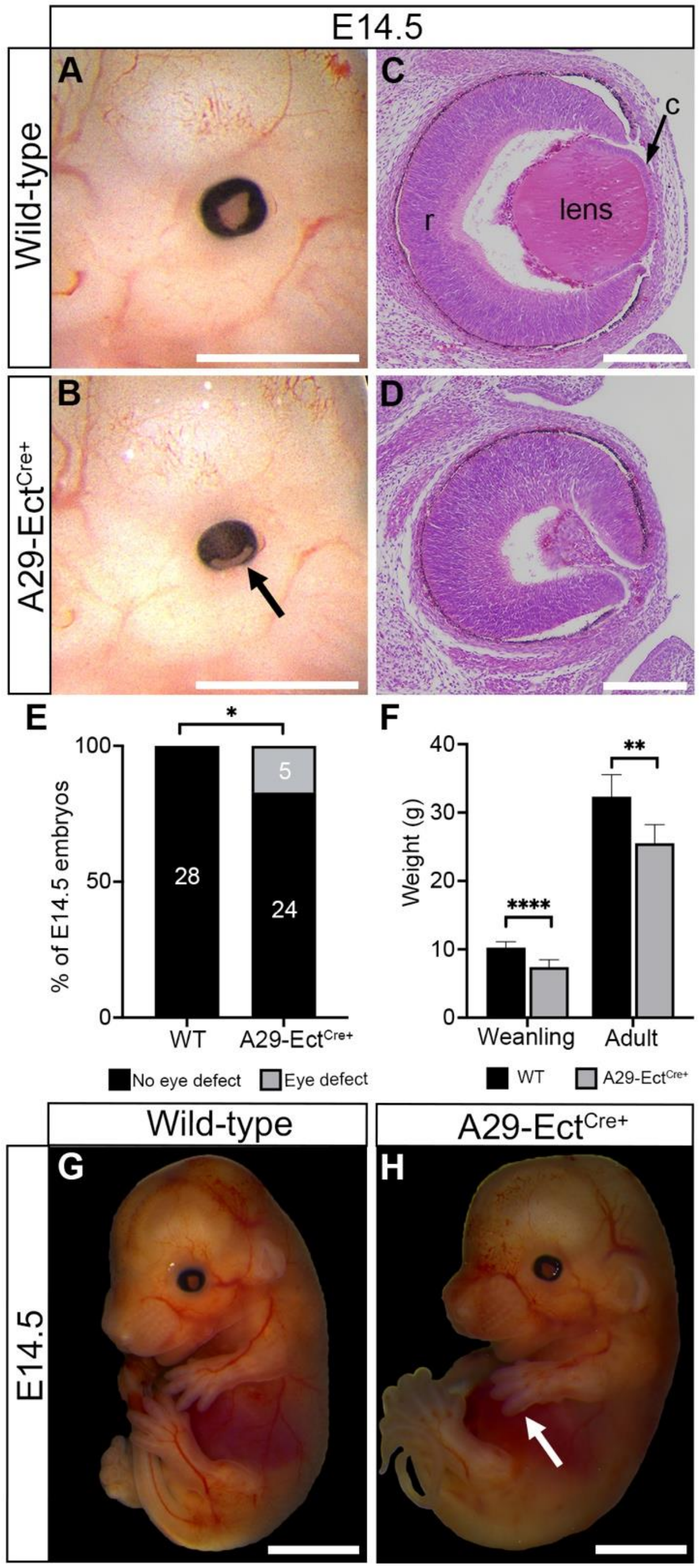

E14.5
A
B
C
D
Wild-type
A29-Ect^Cre+
c
lens
r
E
% of E14.5 embryos
100
50
0
28
24
5
*
WT
A29-Ect^Cre+
No eye defect
Eye defect
F
Weight (g)
40
30
20
10
0
****
**
Weanling
Adult
WT
A29-Ect^Cre+
Wild-type
A29-Ect^Cre+
G
H
E14.5

Fig. S3. Loss of ARHGAP29 leads to ocular and digital defects, and decreased weight. (A-B) Macroscopic images of eyes from wild-type (A) and *Arhgap29*$^{fl/fl}$;*Ect*$^{Cre+}$ (A29-EctCre+, B) E14.5 embryos. Black arrow indicates ocular defect. (C-D) Hematoxylin and Eosin-stained coronal sections of wild-type (C) and *Arhgap29*$^{fl/fl}$;*Ect*$^{Cre+}$ (D) eye at E14.5. (E) Quantification of eye defects in E14.5 embryos. *$P < 0.05$, Chi-square test. (F) Quantification of weanling and adult body weight. ****$P < 0.0001$, **$P < 0.01$ unpaired Student's *t*-test. (G-H) Macroscopic images from wild-type (G) and *Arhgap29*$^{fl/fl}$;*Ect*$^{Cre+}$ (H) E14.5 embryos. White arrow indicates ectrodactyly (missing one digit). c, cornea; r, retina; l, lens. Scale bars: A-D, 200 μm; G-H, 250 μm.

Development • Supplementary information

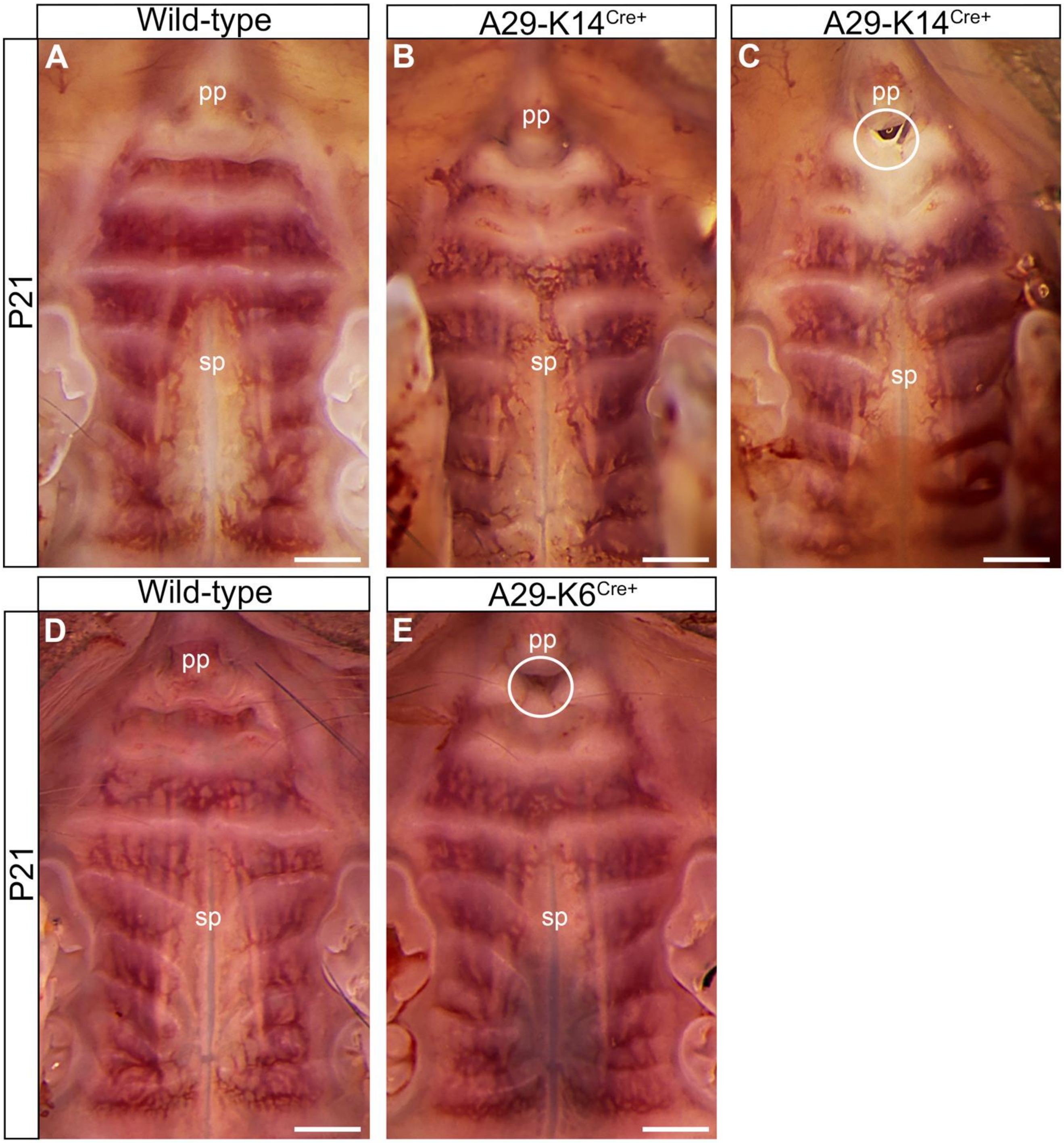

Fig. S4. Loss of ARHGAP29 in basal epithelial or periderm cells only results in an anterior cleft of the secondary palate. (A-E) Macroscopic images of P21 wild-type (A,D), *Arhgap29$^{fl/fl}$;K14^{Cre+}* (A29-K14^{Cre+}, B,C), and *Arhgap29$^{fl/fl}$;K6^{Cre+}* (A29-K6^{Cre+}, E) palates. White circles highlight the anterior cleft defect. pp, primary palate, sp, secondary palate. Scale bars: 500 µm.

Development • Supplementary information

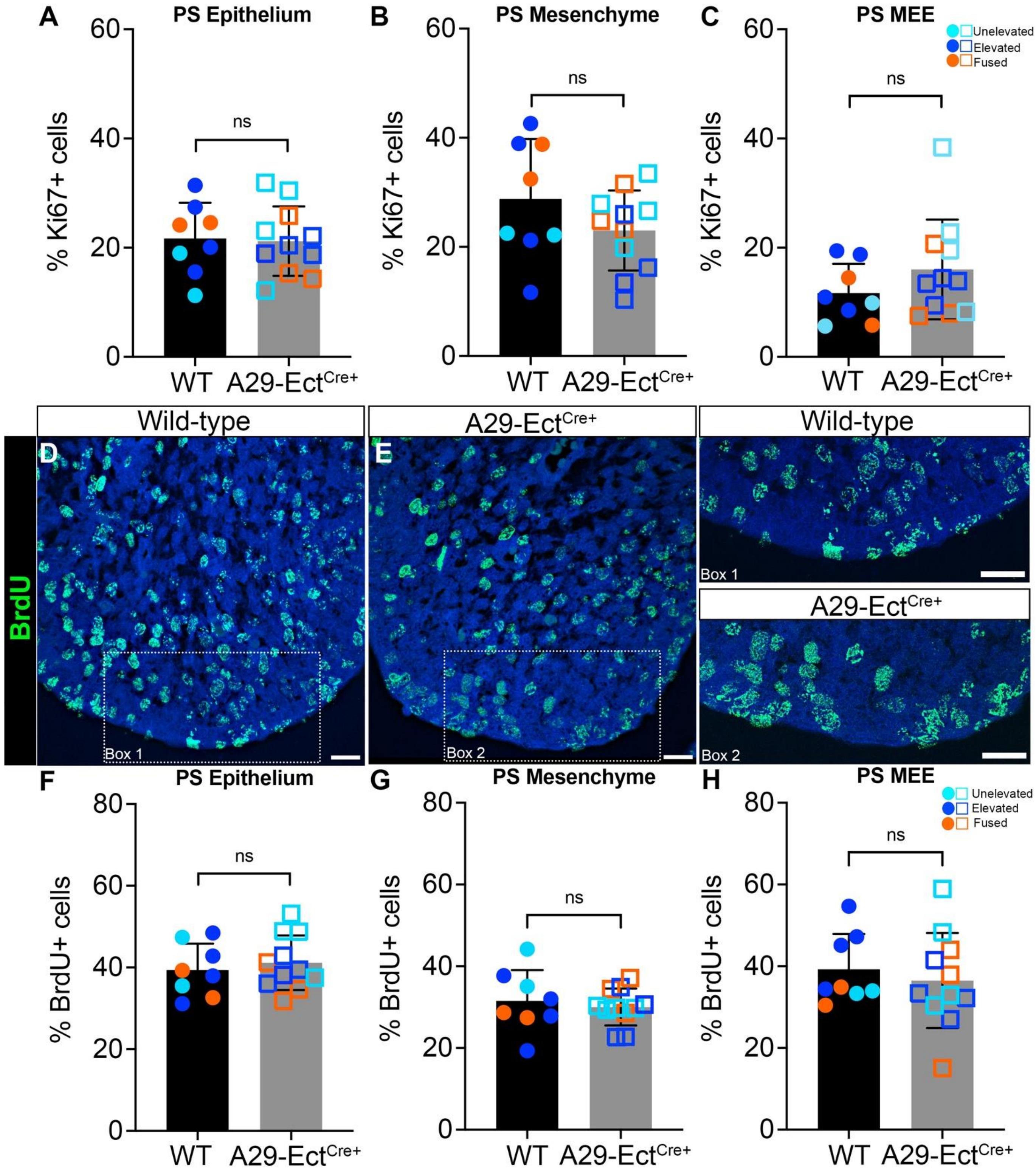
A
PS Epithelium
B
PS Mesenchyme
C
PS MEE
Unelevated
Elevated
Fused
% Ki67+ cells
ns
WT
A29-Ect^{Cre+}
Wild-type
A29-Ect^{Cre+}
D
E
BrdU
Box 1
Box 2
F
PS Epithelium
G
PS Mesenchyme
H
PS MEE
% BrdU+ cells

Fig. S5. ARHGAP29 is not required for proliferation during palatogenesis. (A-C) Quantification of the percentage of Ki-67 positive cells in E14.5 palatal shelf (PS) epithelium (A), mesenchyme (B), and medial edge epithelium (MEE, C). (D-E) Coronal sections of E14.5 wild-type (D, Box 1) and $Arhgap29^{fl/fl};Ect^{Cre+}$ (A29-Ect^{Cre+}, E, Box 2) palatal shelves stained with BrdU (green). Magnified images of panels D and E are shown in Boxes 1 and 2, respectively. (F-H) Quantification of the percentage of BrdU positive cells in E14.5 PS epithelium (F), mesenchyme (G), and MEE (H). ns, not significant following Student's *t*-test. Scale bars: 20 µm

Development • Supplementary information

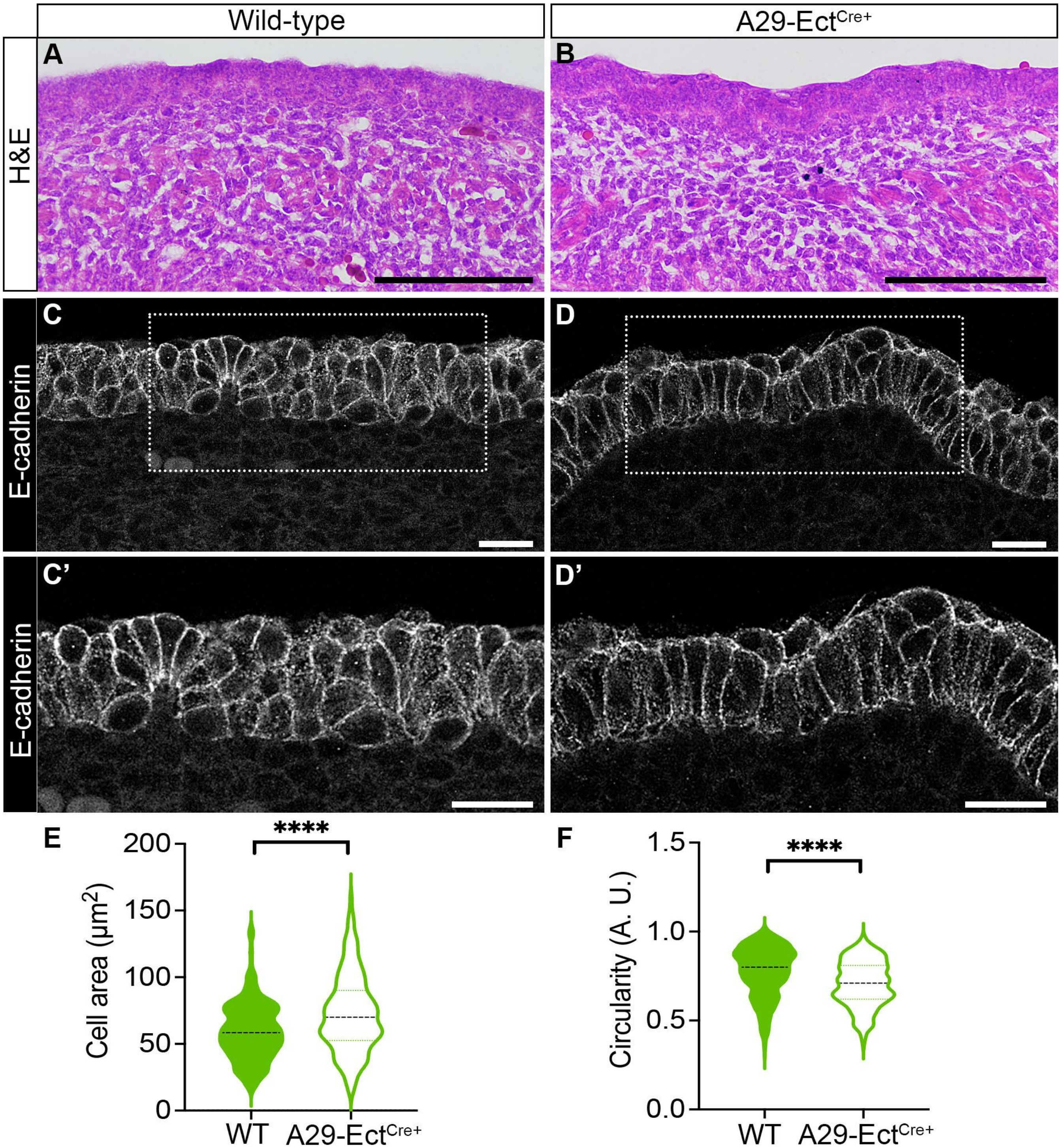

Wild-type
A29-Ect^Cre+
H&E
E-cadherin
E-cadherin
A
B
C
D
C'
D'
E
F
****
Cell area (μm²)
200
150
100
50
0
WT
A29-Ect^Cre+
Circularity (A. U.)
1.5
1.0
0.5
0.0

Fig. S6. *Arhgap29$^{fl/fl}$* ;*Ect^{Cre+}* embryos exhibit a disorganized lingual epithelium. (A,B) Coronal sections of E14.5 tongue from wild-type (A) and *Arhgap29$^{fl/fl}$*;*Ect^{Cre+}* (A29-Ect^{Cre+}, B) embryos stained with Hematoxylin and Eosin. Scale bars: 100 µm. (C-D') Confocal images (single slice) of lingual coronal sections of E14.5 wild-type (C,C') and *Arhgap29$^{fl/fl}$*;*Ect^{Cre+}* (D,D') stained with E-cadherin. Magnified images of panels C and D are shown in panels C' and D', respectively. Scale bars: 20 µm. (E) Quantification of lingual epithelial cell area. (F) Quantification of lingual epithelial cell circularity. ****P < 0.0001, unpaired Student's *t*-test.

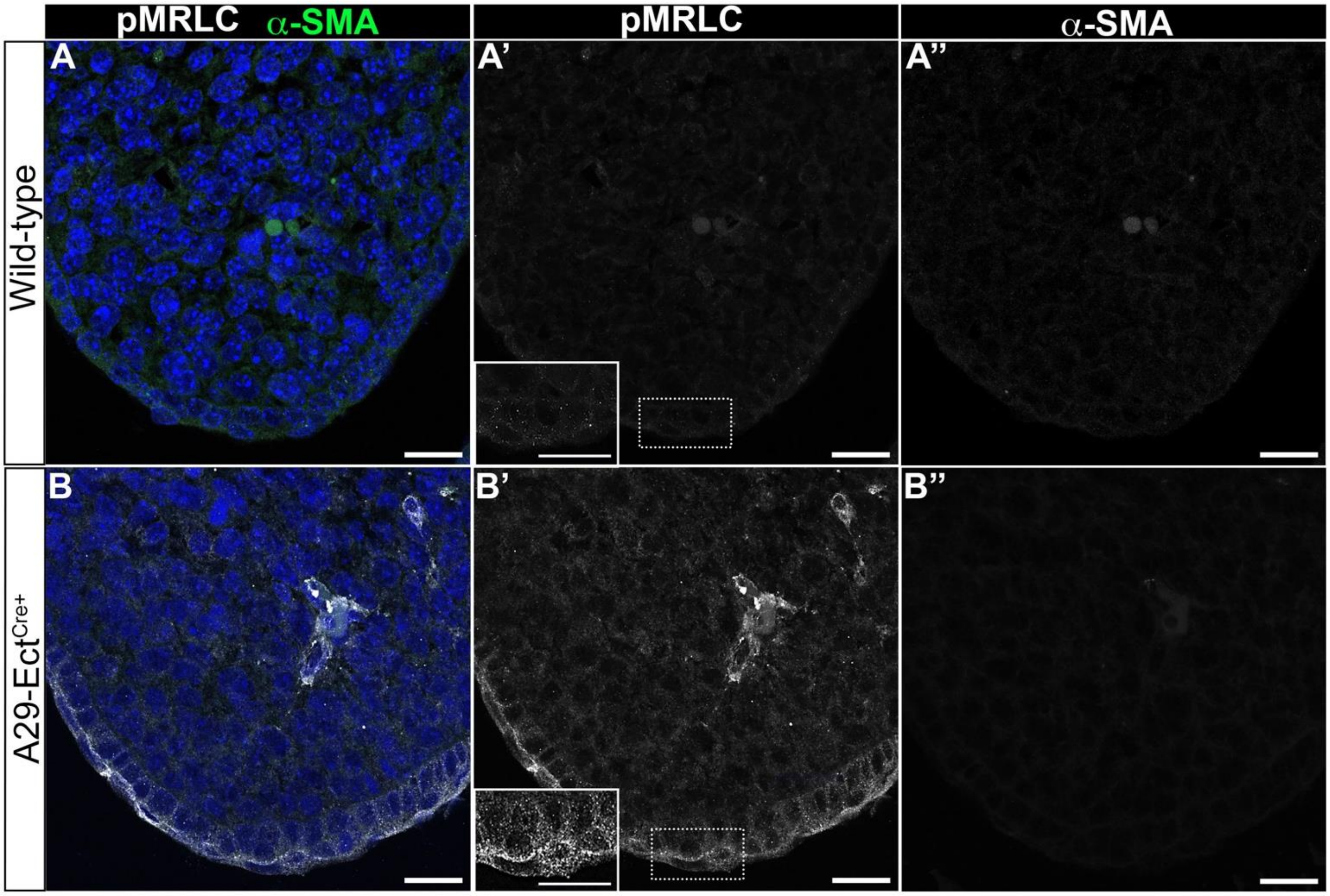

Fig. S7. Loss of ARHGAP29 in basal epithelial and periderm cells increases contractility protein activity. (A-B'') Maximum projections of 10-15 confocal slices of unelevated palatal shelves from E14.5 wild-type (A-A") and *Arhgap29*$^{fl/fl}$;*Ect*$^{Cre+}$ (A29-Ect^{Cre+}, B-B'') embryos dual stained with phospho-myosin regulatory light chain (pMRLC, white in merged) and alpha-smooth muscle actin (α-SMA, green in merged). Scale bars: 20 μm.

Development • Supplementary information

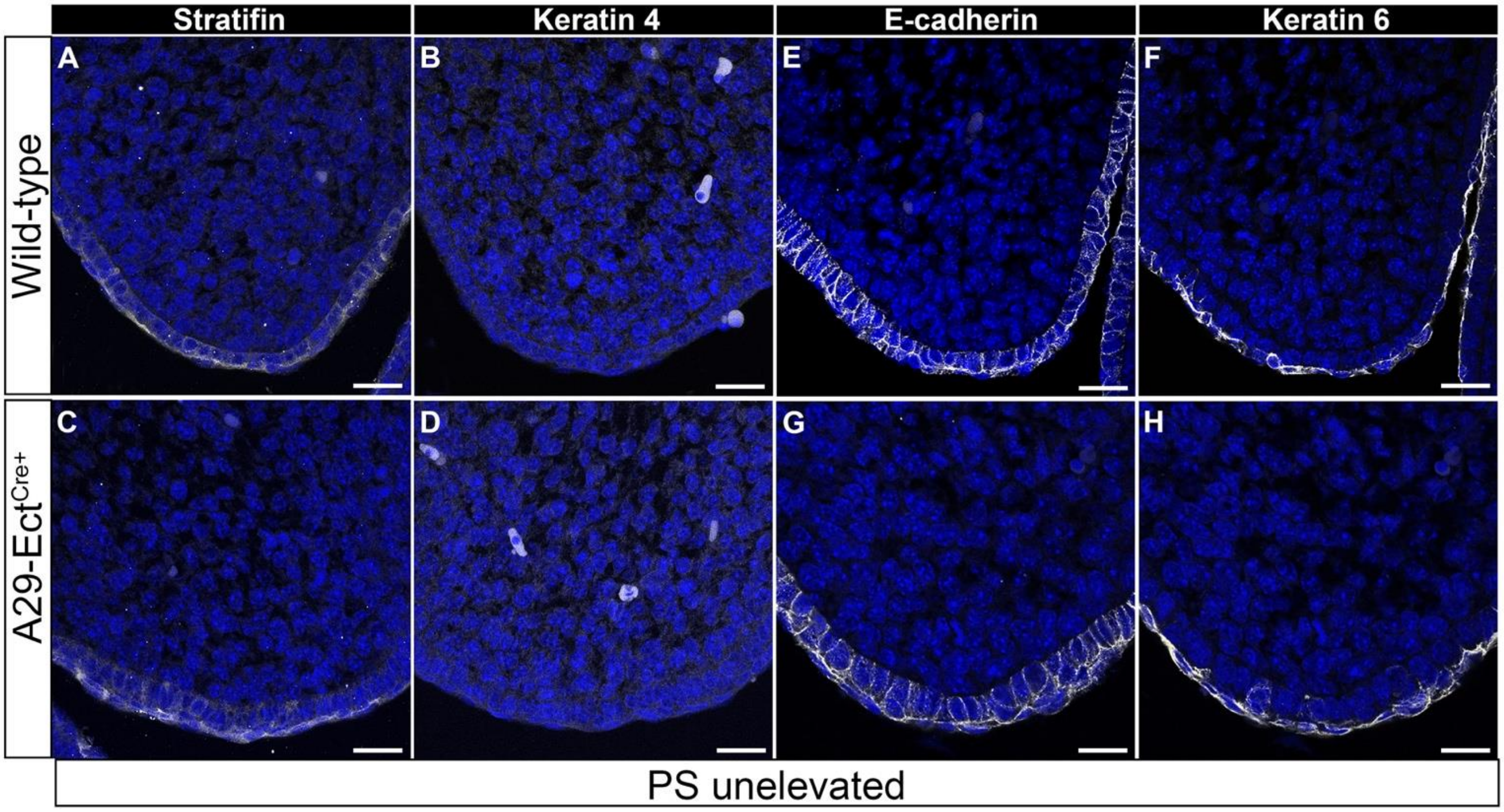

Fig. S8. *Arhgap29$^{fl/fl}$;Ect^{Cre+}* embryos show unchanged epithelial identity and differentiation prior to elevation. (A-H) Maximum projections of two to four confocal slices of unelevated palatal shelves from E14.5 wild-type (A,B,E,F) and *Arhgap29$^{fl/fl}$;Ect^{Cre+}* (A29-Ect^{Cre+}; C,D,G,H) embryos stained with Stratifin (A,C), Keratin 4 (B,D), E-cadherin (E,G), and Keratin 6 (F,H). Scale bars: 20 µm.

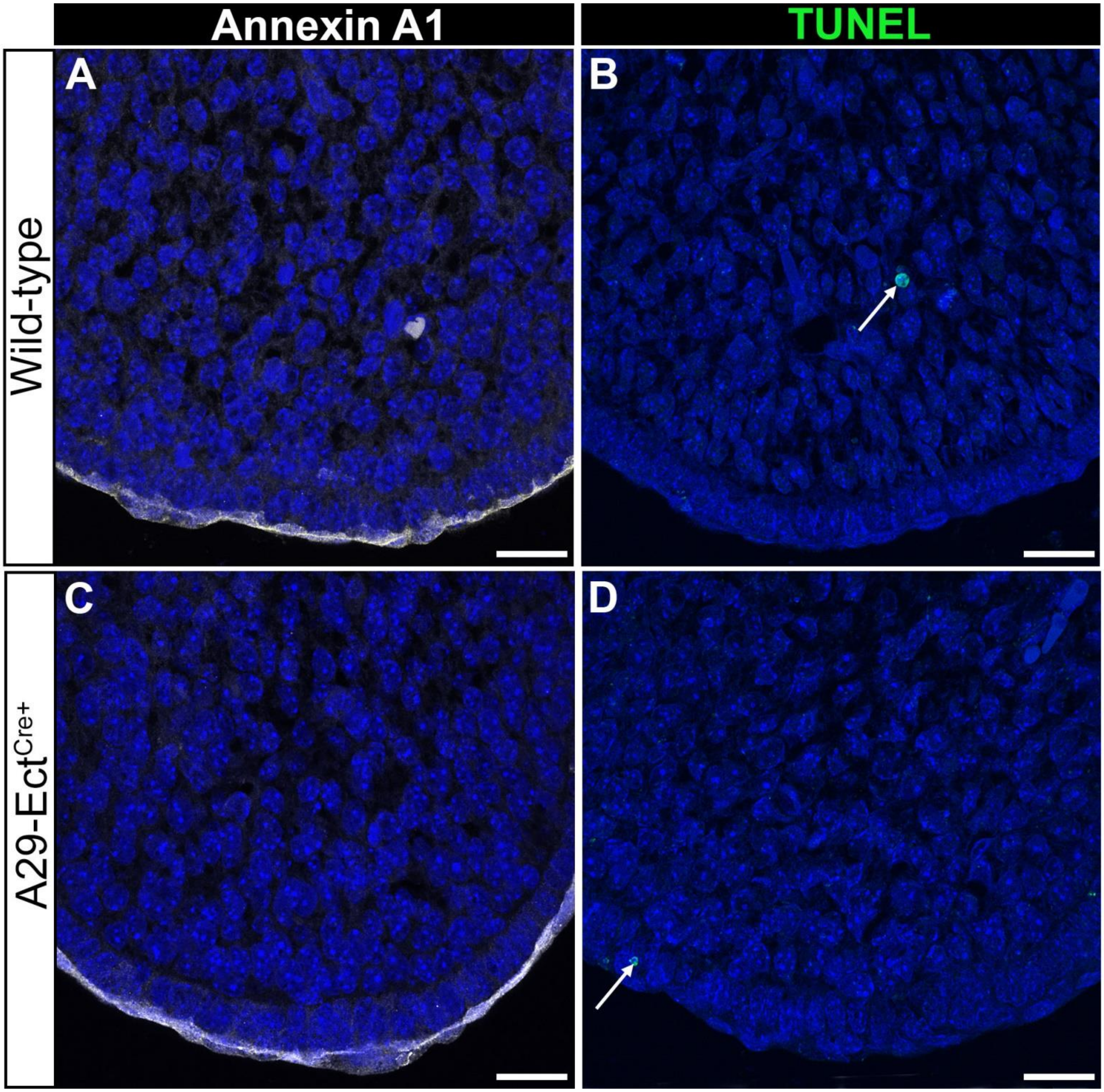

Fig. S9. *Arhgap29-Ect^{Cre+}* embryos show unchanged apoptosis prior to elevation. (A-D) Coronal sections of E14.5 wild-type (A,B) and *Arhgap29$^{fl/fl}$*;*Ect^{Cre+}* (A29-Ect^{Cre+}; C,D) with unelevated palatal shelves stained with Annexin A1 (grey; A,C) or TUNEL (green; B,D). White arrows point to TUNEL positive cells. Scale bars: 20 μm.

Table S1. List of antibodies used in this study

Antibody Name	Host Species	Company Name	Company Location	Catalog #	RRID	Dilution
**Primary Antibodies**						
Alpha-Smooth Muscle Actin	Mouse Monoclonal	Sigma-Aldrich	St. Louis. MO	A2547	RRID:AB_476701	1/8000
Annexin A1	Rabbit Polyclonal	Invitrogen	Waltham, MA	713400	RRID:AB_88080	1/5000
Arhgap29	Rabbit Polyclonal	Novus	Littleton, CO	NBP1-05989	RRID:AB_1556171	1/100
Arhgap29	Rabbit Polyclonal	Proteintech	Rosemont, IL	12583-1-AP	RRID:AB_2918030	1/100
Bromodeoxyuridine	Rat Monoclonal	Abcam	Cambridge, UK	ab6326	RRID:AB_305426	1/100
E-Cadherin	Mouse Monoclonal	BD Biosciences	San Jose, CA	610181	RRID:AB_397580	1/100
GFP	Rabbit Polyclonal	Invitrogen	Waltham, MA	A6455	RRID:AB_2313717	1/400
Keratin 4	Rabbit Polyclonal	Invitrogen	Waltham, MA	MA537810	RRID:AB_2897734	1/3200
Keratin 6	Rabbit Polyclonal	Covance	Princeton, NJ	PRB-169P	RRID:AB_10063923	1/500
Ki-67	Rabbit Monoclonal	Invitrogen	Waltham, MA	RM-9106-S	RRID:AB_2341197	1/200
Phosphorylated Myosin Regulatory Light Chain	Rabbit Polyclonal	Cell Signaling	Danvers, MA	3671	RRID:AB_330248	1/100
Stratifin	Goat Polyclonal	Santa Cruz	Dallas, TX	SC-7683	RRID:AB_2301664	1/100
**Secondary Antibodies**						
Alexa Fluor 488 goat anti-mouse	Mouse	Invitrogen	Waltham, MA	A-11001	RRID:AB_2534069	1/400
Alexa Fluor 488 goat anti-rabbit	Rabbit			A-11008	RRID:AB_143165	1/400
Alexa Fluor 568 goat anti-mouse	Mouse			A-11004	RRID:AB_2534072	1/400
Alexa Fluor 568 goat anti-rabbit	Rabbit			A-11011	RRID:AB_143157	1/400
Alexa Fluor 594 donkey anti-goat	Donkey			A-11058	RRID:AB_142540	1/400
Anti-Rat IgG-FITC	Rat	Chemicon	Rolling Meadows, IL	AP136F	RRID:AB_90876	1/200
