# Supplementary material for: Ectodermal loss of ARHGAP29 alters epithelial morphology and disrupts murine palatogenesis
Source: Development. 2026 Jun 29;153(12):dev205636. doi: 10.1242/dev.205636 (PMC13594415; doi:10.1242/dev.205636)
Supplement: Supplementary file 2 [file develop-153-205636_review_history.pdf]

Development

Ectodermal loss of ARHGAP29 alters epithelial morphology and disrupts murine palatogenesis

Emily Adelizzi, Lindsey Rhea, Campbell Mitvalsky, Samuel Pek, Bethany Dolittle and Martine Dunnwald

DOI: 10.1242/dev.205636

Editor: Liz Robertson

Review timeline

Original submission:	31 December 2024
Editorial decision:	24 February 2025
Resubmission:	4 March 2026
Editorial decision:	7 April 2026
First revision:	29 April 2026
Editorial decision:	18 May 2026
Second revision:	21 May 2026
Accepted:	22 May 2026

Original submission

First decision letter

MS ID#: dev.204627

MS TITLE: The tissue-specific loss of ARHGAP29 disrupts murine palatal development

AUTHORS: Emily Christine Adelizzi, Lindsey Rhea, Campbell Mitvalsky, Samuel Pek, Bethany Dolittle and Martine Dunnwald

Dear Dr Dunnwald,

I have now received all the referees reports on the above manuscript, and have reached a decision. The referees' comments are appended below, or you can access them online: please go to:.

As you will see from their reports, the referees recognise the potential of your work, but they also raise significant concerns about it. Given the nature of these concerns, I am afraid I have little choice other than to reject the paper at this stage.

However, having evaluated the paper, I do recognise the potential importance of this work. I would therefore be prepared to consider as a new submission an extension of this study that contains new experiments, data and discussions and that address fully the major concerns of the referees. The work required goes beyond a standard revision of the paper. Please bear in mind that the referees (who may be different from the present reviewers) will assess the novelty of your work in the context of all previous publications, including those published between now and the time of resubmission.

Reviewer 1: SUMMARY OF THE ADVANCE MADE IN THIS PAPER AND ITS POTENTIAL SIGNIFICANCE TO THE FIELD

© 2026. Published by The Company of Biologists under the terms of the Creative Commons Attribution License (https://creativecommons.org/licenses/by/4.0/).

Mutations in ARHGAP29 are associated with cleft palate in humans, but it was unknown how ARHGAP29 contributes to palate development. Adelizzi et al. provide the first evidence that Arhgap29 is required in the ectoderm for palate shelf elevation and fusion. Furthermore, they show that arhgap29 is required for the organization of the epithelium at the tip of teh shelves and for the organization of the alpha-smooth muscle actin and phospho-myosin regulatory light chain in the palatal epithelium. Furthermore, supplementary data suggest that Arhgap29 is required for lingual and eye development as well.

SUGGESTIONS TO AUTHORS

The data presented are clear and convincing. I have some suggestions for improving the clarity and flow of the manuscript.

I followed paragraph 1 and paragraph 2 well: Generation and validation of Arhgap29 conditional knockouts and A29-EctCre+ embryos display gross morphological defects. I am convinced by these data and paragraphs that the authors h ave the tools they need to answer the posed questions. After this, I suggest moving straight to A29-Ect Cre+ palate data.

I suggest leading with the positive data, which is the most interesting. Thus, after the introductory characterization of A29-Ect Cre+, A29-Ect Cre+ exhibit defective palatogenesis and cleft palate— you can clarify this subtitle as well by changing the subtitle to what you want your reader to remember: ARHGAP29 is required in X cells for palate formation at y stage.

Start the paragraph with a topic sentence: To determine if ARHGAP29 is required in ectodermal cells, we performed histological analysis of coronal sections of A29-EctCre+ and control palates at E14.5 and E18.5. After the rest of that paragraph, tell your reader about K14 Cre negative data and what the difference between these loss of ARHGAP29 using these two different drivers means. I need the final sentence of the K14 cre A29 to say something like, Because A29-Ect Cre+ have palatal defects and K14 A29 Cre + do not, we conclude that ARHGAP29 is required in the ectoderm and the mesechyme at Y stage and that ARHGAP29 ko from the ectoderm alone is not sufficient to disrupt palate development. Did I get that right?

Paragraph: ARHGAP29 in oral epithelial cells only is not required for proper palatogenesis-- You could clarify this subtitle to "Specific loss of ARHGAP29 from the epithelial cells does not disrupt palatogenesis"

The first sentence needs to include what the authors are testing- To evaluate the requirement for ARHGAP29 in only the epithelial cells of the palate, …..we harvested x genotype of embryos at E14.5 and compared them to Y controls with coronal histological analysis. Again, readers need something at the end of this paragraph to reinstate what the difference is between K14 and A29-Ect Cre+ to help them interpret why one has phenotypes and the other doesn't. Call out differences in timing of the cre drivers as well.

Now, once we know more about the differences between the drivers, I think the authors are saying that the reason A29-Ect Cre+ have phenotypes and A29-K14 Cre+ do not is that A29 is knocked down in the mesenchyme and is knocked down a bit earlier with the Ect-Cre. So now, the authors can dig into the mechanisms. I appreciate the model figure.

The discussion is well written.

Again, the data is interesting and well presented. My suggestions are that the organization of the results be rearranged to help the flow and emphasize the conclusions that can be drawn from the data.

Reviewer 2: SUMMARY OF THE ADVANCE MADE IN THIS PAPER AND ITS POTENTIAL SIGNIFICANCE TO THE FIELD

The manuscript by Adelizzi et al describes the analysis of conditional knockout of Arhgap29, a gene contributing to risk of cleft lip/palate in humans. The authors have previously described a global

© 2026. Published by The Company of Biologists under the terms of the Creative Commons Attribution License (https://creativecommons.org/licenses/by/4.0/).

knockout of the gene, reporting occasional ectopic epithelial adhesions in heterozygotes, whereas homozygotes were early embryonic lethal. In this manuscript the authors use two different epithelial-specific Cre drivers: K14-Cre which turns on in basal epithelia after E11.5 (after primary palatal development but before secondary palatogenesis), and Ect-Cre (Crect) which begins expressing Cre in all epithelia by E8.5. Although not clearly indicated, it appears the authors are describing the phenotype in conditional homozygotes only. They do not mention whether any ectopic adhesions were observed in conditional heterozygotes which would have been useful to resolve the cell-specific origin of those "biomarkers" of CL/P. Instead, the authors focus on the penetrance and nature of secondary palatal clefting, which they note only in the Ect-Cre conditional embryos. They note oral epithelial changes (hyperplasia) in mutants that begins on the anterior palatal and oral region before being found more posteriorly as the palatal shelves elevate. The authors then assess the observed epithelial hyperplasia using standard markers and both BrdU and TUNEL labeling. They conclude that the palatal phenotype is due to changes in cytoskeletal contractility, although no direct evidence is provided.

SUGGESTIONS TO AUTHORS

Overall, the work is a useful but not a markedly significant extension to their prior studies. However, there are numerous issues that reduce the significance and impact of the presented data.

1. The title does not reflect the conclusions of the paper in that epithelial origin should be mentioned.
2. The Abstract discusses cleft palate, yet the Introduction highlights that ARHGAP29 is implicated in cleft lip/palate in human studies. Further, the authors do not investigate the epithelia of the fusing lip, even though the Ect-Cre driver used is active at that time in that tissue.
3. Epithelial hyperplasia has been seen previously in other genes implicated in CL/P. These are barely mentioned nor compared to the results in the conditional Arhgap29 mice.
4. There is no mention of whether ectopic adhesions are seen in heterozygotes as with their global knockout heterozygotes. If there aren't, why?
5. The conclusion that contractility is impacted is reasonable but is based solely on immunohistochemical staining at one embryonic age. It has certainly not been demonstrated as suggested by the authors. The authors have previously used Arhgap29 deficient keratinocytes which would be a useful source to verify their suggested mechanism.
6. The authors should clearly indicate that the embryos being analyzed are homozygotes, with the full genotype presented. Also, while it is reasonable to use the "A29" abbreviation in figures, A29 is not an approved gene abbreviation and should not be used in the body of the manuscript.
7. It has previously been shown that Ect-Cre requires transmission through the male germline to maintain epithelial-specific expression. While it states that embryos were generated using Ect-Cre positive males, one wonders if at some point Cre passed through the female germline. Because the authors raise questions about the previously described epithelial-specificity of this Cre driver, the authors should make it clear if they have never taken it through the female germline and how often they test the specificity of the driver.

The authors should check the manuscript for instances where the gene or allele name has not been italicized when it should. Also, when referring to the mouse gene, only the first letter should be italicized - unlike human genes, which are fully capitalized.

No information on the number of embryos with syndactyly and edema is presented. Ocular anomalies like those presented are seen occasionally in C57Bl/6J animals, so it is not clear whether these are specific to Arhgap29 knockout. Demonstration that the gene is expressed in the eyes and there are appropriate cellular changes consistent with that expression as they have done with the palate.

It is unclear how the authors reconcile the phenotypes seen at E14.5 (where most embryos appear to have just anterior and posterior clefts and touching in the middle, to the percentages of full cleft palate seen at E18.5.

The authors mention Fig 3U, but it should read Fig 3J.

© 2026. Published by The Company of Biologists under the terms of the Creative Commons Attribution License (https://creativecommons.org/licenses/by/4.0/).

The graphs in Fig 4 (especially P, R and S) are so compact, it is difficult to appreciate any difference in means, even though the differences are supposedly highly significant. Why is the p value shown in Fig 4R but asterisks are shown in the other graphs?

The reference to 20% palatal clefting being considered substantial for a single gene contribution is misleading. There are many genes that in a homozygous null state result in a much higher percentage of cleft palate. 20% would be significant is seen in heterozygotes but this does not appear to be the case.

When referring to beta-catenin, SPECC1L and Nectins, the authors should use italicization. Further, as a mouse gene it should be written Specc1l, and the authors should be more precise about which Nectin, ie. Nectin1.

It was strange for the authors to refer to the Tbx1 example when the Irf6 example would have been more relevant.

An increase in size cannot explain hyperplasia as, by definition, hyperplasia is an increase in cell number.

The 'flip up' process originally proposed by Coleman in 1965 has been studied many times in recent decades with much support for the hypothesis. These latter studies would be worth citing given they provide some experimental support.

The studies do not show that both the basal and periderm are required. The K14-Cre indeed shows that the basal epithelia are not sufficient. But the Ect-Cre cannot rule out that only the periderm is required as both are affected. If the authors used a periderm-specific Cre, then they could draw such a definitive conclusion.

Like gene names, the different Cre alleles should also be italicized. The source of the Cre drivers should also be listed or acknowledged.

The Methods sections - Histological analysis and phenotype scoring (first three sentences) and Fluorescent immunostaining and microscopy (first sentence) - need to be rewritten as the grammar is poor.

The authors state that the conditional Arhgap29 animals lose weight following weaning. However, data is only shown (Fig S2) for animals at weaning, and already their weight is low. If weights were taken at other ages then this should be shown. If not, the authors need to change the conclusions - it would seem that the conditional null animals have reduced weight at weaning, but perhaps not through weight loss.

It has previously been suggested that ectopic adhesions arise because of changes in E-cadherin (or other adhesion proteins) on the periderm. Are there any quantitative differences in apical E-cadherin staining in mutants vs controls?

Reviewer 3: SUMMARY OF THE ADVANCE MADE IN THIS PAPER AND ITS POTENTIAL SIGNIFICANCE TO THE FIELD

This manuscript reports analysis of the function of Arhgap29 in palate development using a conditional knockout approach in mice. The authors report that early ectodermal Cre-mediated deletion of Arhgap29, but not K14-Cre mediated oral epithelial specific deletion of Arhgap29, caused a partial but significant penetrance of cleft palate associated with changes in palatal epithelial morphology and expression of markers of cell contractility. The authors conclude this study provides the first direct evidence that ARHGAP29 is essential for proper palatal elevation and fusion.

SUGGESTIONS TO AUTHORS

Several issues need to be resolved to support the authors' conclusions:

© 2026. Published by The Company of Biologists under the terms of the Creative Commons Attribution License (https://creativecommons.org/licenses/by/4.0/).

1. The title is vague regarding ARHGAP20 function. A more appropriate title could be "ARHGAP29 plays an important role in palatal epithelial organization during palatogenesis".
2. The description and analysis of the Arhgap29 conditional allele are not sufficiently informative. Previous studies suggested Arhgap29 heterozygosity caused oral epithelial adhesions, but no such phenotype is observed in either the K14-Cre cko or the Ect-Cre cko embryos [by the way, the Ect-Cre strain should be described using official nomenclature Hhat>Tg(TFAP2A-cre)1Will) when first appeared in the text], raising a question whether the Cre-mediated recombination of the A29fl allele could completely inactivate its function. The manuscript describes the A29fl allele as a FLAG tagged flossed Arhgap29 allele generated at the University of Iowa Core Facility. Figure 1A shows a schematic with loxP sites in introns 3 and 5. It is not clear whether deletion of exons 4 and 5 results in a null allele. There is no description whether germline or early embryonic deletion of the A29fl allele recapitulates the previous A29 homozygous knockout phenotype. Or whether the previous nonsense allele might have a dominant negative effect to account for the oral epithelial adhesion defects observed.
3. As the manuscript showed, the Ect-Cre has Cre expression/activity in the palatal mesenchyme. The authors indicated that the A29-Ect-cre should be considered a combined mesenchyme knockdown/ectoderm knockout rather than a strict ectoderm-specific conditional murine model. The lack of phenotype of K14-Cre cko together with the presence of significant mesenchymal Cre activity and incomplete penetrance of cleft palate in the Ect-Cre cko mutants raise obvious questions whether Arhgap29 function is required in the palatal epithelium for palate development and whether the variability in cleft palate could primarily be due to the incomplete knockdown in the palatal mesenchyme or other nonpalatal structures. The manuscript subheading of a results section as "ARHGAP29 in oral epithelial cells only is not required for proper palatogenesis" and describing the Ect-Cre cko palate defect as "secondary cleft palate" further confuse the matter. While the data presented provide solid evidence for changes in palatal epithelial morphology, organization, and expression of contractility markers, additional studies are needed to elucidate whether those changes are due to loss of A29 in the palatal epithelium or mesenchyme or both.
4. The alterations in epithelial morphology, organization, and marker expression are interesting, but whether and how they correlate with cleft palate are not clear. More careful analysis of embryos at E13.5 to E14.5, prior to palatal shelf fusion in both the control and mutant embryos, should be analyzed and scored to see how the changes are consistent with genotypes. If all mutant embryos exhibit consistent epithelial changes, then why only ~23% mutant embryos showed cleft palate?

First Resubmission

Response to Original Reviews

Reviewer 1: SUMMARY OF THE ADVANCE MADE IN THIS PAPER AND ITS POTENTIAL SIGNIFICANCE TO THE FIELD

Mutations in ARHGAP29 are associated with cleft palate in humans, but it was unknown how ARHGAP29 contributes to palate development. Adelizzi et al. provide the first evidence that Arhgap29 is required in the ectoderm for palate shelf elevation and fusion. Furthermore, they show that arhgap29 is required for the organization of the epithelium at the tip of the shelves and for the organization of the alpha-smooth muscle actin and phospho-myosin regulatory light chain in the palatal epithelium. Furthermore, supplementary data suggest that Arhgap29 is required for lingual and eye development as well.

SUGGESTIONS TO AUTHORS

The data presented are clear and convincing. I have some suggestions for improving the clarity and flow of the manuscript.

I followed paragraph 1 and paragraph 2 well: Generation and validation of Arhgap29 conditional knockouts and A29-EctCre+ embryos display gross morphological defects. I am convinced by these data and paragraphs that the authors have the tools they need to answer the posed questions. After this, I suggest moving straight to A29-Ect Cre+ palate data.

© 2026. Published by The Company of Biologists under the terms of the Creative Commons Attribution License (https://creativecommons.org/licenses/by/4.0/).

I suggest leading with the positive data, which is the most interesting. Thus, after the introductory characterization of A29-Ect Cre+, A29-Ect Cre+ exhibit defective palatogenesis and cleft palate—you can clarify this subtitle as well by changing the subtitle to what you want your reader to remember: ARHGAP29 is required in X cells for palate formation at y stage.

Start the paragraph with a topic sentence: To determine if ARHGAP29 is required in ectodermal cells, we performed histological analysis of coronal sections of A29-EctCre+ and control palates at E14.5 and E18.5. After the rest of that paragraph, tell your reader about K14 Cre negative data and what the difference between these loss of ARHGAP29 using these two different drivers means. I need the final sentence of the K14 cre A29 to say something like, Because A29-Ect Cre+ have palatal defects and K14 A29 Cre + do not, we conclude that ARHGAP29 is required in the ectoderm and the mesechyme at Y stage and that ARHGAP29 ko from the ectoderm alone is not sufficient to disrupt palate development. Did I get that right?

Paragraph: ARHGAP29 in oral epithelial cells only is not required for proper palatogenesis-- You could clarify this subtitle to "Specific loss of ARHGAP29 from the epithelial cells does not disrupt palatogenesis"

The first sentence needs to include what the authors are testing- To evaluate the requirement for ARHGAP29 in only the epithelial cells of the palate, …..we harvested x genotype of embryos at E14.5 and compared them to Y controls with coronal histological analysis. Again, readers need something at the end of this paragraph to reinstate what the difference is between K14 and A29-Ect Cre+ to help them interpret why one has phenotypes and the other doesn't. Call out differences in timing of the cre drivers as well.

Now, once we know more about the differences between the drivers, I think the authors are saying that the reason A29-Ect Cre+ have phenotypes and A29-K14 Cre+ do not is that A29 is knocked down in the mesenchyme and is knocked down a bit earlier with the Ect-Cre. So now, the authors can dig into the mechanisms. I appreciate the model figure.

We appreciate the great suggestions of this reviewer. As our revised manuscript contains additional data, the flow of the revised manuscript has changed substantially. We took into account this reviewer's suggestion in the flow of our revised results.

The discussion is well written.

Again, the data is interesting and well presented. My suggestions are that the organization of the results be rearranged to help the flow and emphasize the conclusions that can be drawn from the data.

We have addressed these comments in the manuscript to improve the organization and clarity

---

Reviewer 2: SUMMARY OF THE ADVANCE MADE IN THIS PAPER AND ITS POTENTIAL SIGNIFICANCE TO THE FIELD

The manuscript by Adelizzi et al describes the analysis of conditional knockout of Arhgap29, a gene contributing to risk of cleft lip/palate in humans. The authors have previously described a global knockout of the gene, reporting occasional ectopic epithelial adhesions in heterozygotes, whereas homozygotes were early embryonic lethal. In this manuscript the authors use two different epithelial-specific Cre drivers: K14-Cre which turns on in basal epithelia after E11.5 (after primary palatal development but before secondary palatogenesis), and Ect-Cre (Crect) which begins expressing Cre in all epithelia by E8.5. Although not clearly indicated, it appears the authors are describing the phenotype in conditional homozygotes only. They do not mention whether any ectopic adhesions were observed in conditional heterozygotes which would have been useful to resolve the cell-specific origin of those "biomarkers" of CL/P. Instead, the authors focus on the penetrance and nature of secondary palatal clefting, which they note only

© 2026. Published by The Company of Biologists under the terms of the Creative Commons Attribution License (https://creativecommons.org/licenses/by/4.0/).

in the Ect-Cre conditional embryos. They note oral epithelial changes (hyperplasia) in mutants that begins on the anterior palatal and oral region before being found more posteriorly as the palatal shelves elevate. The authors then assess the observed epithelial hyperplasia using standard markers and both BrdU and TUNEL labeling. They conclude that the palatal phenotype is due to changes in cytoskeletal contractility, although no direct evidence is provided.

SUGGESTIONS TO AUTHORS

Overall, the work is a useful but not a markedly significant extension to their prior studies. However, there are numerous issues that reduce the significance and impact of the presented data.

1. The title does not reflect the conclusions of the paper in that epithelial origin should be

We have modified our title to better reflect the conclusions of our studies. The new title reads “The ectodermal loss of ARHGAP29 alters epithelial morphology and disrupts murine palatal development”

2. The Abstract discusses cleft palate, yet the Introduction highlights that ARHGAP29 is implicated in cleft lip/palate in human studies. Further, the authors do not investigate the epithelia of the fusing lip, even though the Ect-Cre driver used is active at that time in that tissue.

We have modified the text to address this comment. The text now reads “None of the murine models generated displayed the presence of a cleft lip”

3. Epithelial hyperplasia has been seen previously in other genes implicated in CL/P. These are barely mentioned nor compared to the results in the conditional Arhgap29 mice.

We have addressed this comment by clarifying the nature of our epithelial phenotype including more information in the discussion.

4. There is no mention of whether ectopic adhesions are seen in heterozygotes as with their global knockout heterozygotes. If there aren't, why?

We clarified in our manuscript that for our studies, we only generated heterozygous mutants to evaluate viability, then we switched breeding schemes to only generate homozygous mutants. Therefore, we never characterized heterozygous animals for any of the phenotypes we analyzed.
Regarding the oral adhesions, we changed the text to describe how we analyzed them. We also elaborated on our results. Our revised text now reads “To our surprise, we did not observe oral adhesions in Arhgap29-K14^{Cre+}, Arhgap29-K6^{Cre+} or Arhgap29-Ect^{Cre+} homozygotes (data not shown).
The lack of oral adhesions in the Arhgap29-K14^{Cre+} can likely be explained by the fact that ARHGAP29 is still present in the periderm. However their absence in the Arhgap29-K6^{Cre+} and Arhgap29-Ect^{Cre+} are more puzzling. Because the loss-of-function heterozygous model led to a 50% reduction of ARHGAP29 from time of conception, and the changes in ARHGAP29 levels in Arhgap29-Ect^{Cre+} mutants occurred mid-gestation, one may speculate that there may be a non-cell autonomous temporal requirement for ARHGAP29 function in the periderm. Additionally, our previous loss-of-function heterozygous model was in a full C57BL6/J background, while the murine models described here have mixed genetic backgrounds, suggesting that the more complex genetic background of the Ect-Cre driver may also contribute to this divergent phenotype”.

5. The conclusion that contractility is impacted is reasonable but is based solely on immunohistochemical staining at one embryonic age. It has certainly not been demonstrated as suggested by the authors. The authors have previously used Arhgap29 deficient keratinocytes

© 2026. Published by The Company of Biologists under the terms of the Creative Commons Attribution License (https://creativecommons.org/licenses/by/4.0/).

which would be a useful source to verify their suggested mechanism.

We agree with the reviewer that we have not shown direct evidence of a functional change in contractility and revised carefully our manuscript to not overstate our conclusions. We also provide new data that include multiple palatal positions (unelevated, elevated not fused, fusing) corresponding to increasing developmental timepoints. These data demonstrate changes in contractility markers as palatogenesis progress, further supporting evidence, yet no direct evidence, for a role in contractility in this process. We have also cited our *in vitro* studies because our findings with Arhgap29-deficient keratinocytes further support for our *in vivo* observations. We agree with the reviewer that these cells could be a useful source to verify our mechanism, yet refrain to do so in this manuscript as the study focused on *in vivo* contribution of Arhgap29 to palatogenesis.

6. The authors should clearly indicate that the embryos being analyzed are homozygotes, with the full genotype presented. Also, while it is reasonable to use the "A29" abbreviation in figures, A29 is not an approved gene abbreviation and should not be used in the body of the manuscript.

We have modified our manuscript to specify that homozygous embryos are being analyzed and included the proper gene abbreviations for Arhgap29 throughout the text.

7. It has previously been shown that Ect-Cre requires transmission through the male germline to maintain epithelial-specific expression. While it states that embryos were generated using Ect-Cre positive males, one wonders if at some point Cre passed through the female germline. Because the authors raise questions about the previously described epithelial-specificity of this Cre driver, the authors should make it clear if they have never taken it through the female germline and how often they test the specificity of the driver.

We have specified in the text that we only used Cre positive males for breeding and have not passed on the Cre through females. The text in the methods now reads "Female mice homozygous for the FLAG tagged floxed *Arhgap29* allele (Arhgap29^{fl}; generated at the University of Iowa Core Facility) and for the YFP Rosa-26 allele (ROSA^{yfp} Jax strain #006148) were bred with Arhgap29$^{fl/fl}$; ROSA$^{yfp/yfp}$;Cre-positive males, harboring either Keratin-14 Cre (Tg(Krt14-cre)1Efu), Keratin-6 Cre (Krt6a$^{em1(icre)Vk}$) or *Ect*^{Cre} (Tg(Tcfap2a-cre)1Wil) alleles". Further, we validated the Crect expression pattern on many mutants across multiple litters.

The authors should check the manuscript for instances where the gene or allele name has not been italicized when it should. Also, when referring to the mouse gene, only the first letter should be italicized - unlike human genes, which are fully capitalized.

We verified gene and allele names in our revised manuscript to insure proper format.

No information on the number of embryos with syndactyly and edema is presented. Ocular anomalies like those presented are seen occasionally in C57Bl/6J animals, so it is not clear whether these are specific to Arhgap29 knockout. Demonstration that the gene is expressed in the eyes and there are appropriate cellular changes consistent with that expression as they have done with the palate.

Although we noticed edema, this was difficult to rigorously assess and therefore was removed from the revised manuscript. We clearly observed 2 embryos with ectrodactyly. This number are now provided in our revised manuscript. Regarding the ocular defects, we attempted to identify ocular structures where Arhgap29 is present. Because of high level of background in the eye, we can't be assertive about Arhgap29 in this organ. However, we performed serial coronal sections of embryonic head and show, by histology, an atrophic lens and a phenotype resembling coloboma. These data are presented in Fig. S3 C,D. We find that these ocular anomalies are only present in Arhgap29-Ect^{Cre+} mutants, despite the other 3 mouse models characterized in this study also being of partial C57 background. No such ocular defects were

© 2026. Published by The Company of Biologists under the terms of the Creative Commons Attribution License (https://creativecommons.org/licenses/by/4.0/).

observed in any of the wild-types analyzed, suggesting that this is not due to random C57 background effect. It is worth noting that the recently described CRISPR knockout of Arhgap29 also exhibit eye defects (PMID 40429791) and that Arhgap29 mediates uveal melanoma in GNAQ/11-mutant, supporting evidence for a role for Arhgap29 in ocular morphogenesis and homeostasis (PMID 38486999).

It is unclear how the authors reconcile the phenotypes seen at E14.5 (where most embryos appear to have just anterior and posterior clefts and touching in the middle, to the percentages of full cleft palate seen at E18.5.

We provide two potential explanations for our observations; they are solely based on “static” data as we are unable to perform longitudinal studies of embryos and their palatal development.
At E14.5 in the anterior, middle, and posterior, ~15% of these embryos display palatal shelves that are not elevated, representing a significant delay in palatogenesis whereas palatal shelves that are elevated represent a more moderate delay. We would suspect that the embryos with the most severe delay in palatogenesis (ie, shelves that are down at E14.5) would be the embryos that display cleft at E17.5. Whereas embryos that have palatal shelves elevated, but not fuse, might be able to ‘catch up’ to eventually complete fusion.
Another explanation would be that at E14.5, 21% of embryos have delays in palatogenesis (including shelves not elevated or not fused) in the middle specifically. This could suggest that this pool of embryos that accounts for 21% of the total embryos at E14.5 would be the ones that would develop cleft palate if let to live.
We revised our discussion to provide some of these elements of explanation.

The authors mention Fig 3U, but it should read Fig 3J.

We have modified out text to resolve this error.

The graphs in Fig 4 (especially P, R and S) are so compact, it is difficult to appreciate any difference in means, even though the differences are supposedly highly significant. Why is the p value shown in Fig 4R but asterisks are shown in the other graphs?

We have modified figure 5 (previously figure 4) to improve the size and clarity of the graphs. We removed the p value and show significance or absence of significance using convential abbreviations.

The reference to 20% palatal clefting being considered substantial for a single gene contribution is misleading. There are many genes that in a homozygous null state result in a much higher percentage of cleft palate. 20% would be significant is seen in heterozygotes but this does not appear to be the case.

This reviewer is correct about the 20% in heterozygous. We have modified our text and included other examples of mouse models with a partially penetrant cleft palate.

When referring to beta-catenin, SPECC1L and Nectins, the authors should use italicization. Further, as a mouse gene it should be written Specc1l, and the authors should be more precise about which Nectin, ie. Nectin1.

We have changed the gene names to reflect proper nomenclature. We also specify which Nectin (Nectin 1 and Nectin 4).

It was strange for the authors to refer to the Tbx1 example when the Irf6 example would have been more relevant.

Tbx1 was referred to in the text due to the similarities in thickening of the oral epithelium in Tbx1 mutant embryos, whereas Irf6 mutants have only been shown to have a thickened surface epithelium and no such changes have been described in the oral cavity, due to severe oral

© 2026. Published by The Company of Biologists under the terms of the Creative Commons Attribution License (https://creativecommons.org/licenses/by/4.0/).

fusion observed in Irf6 deficient embryos.

An increase in size cannot explain hyperplasia as, by definition, hyperplasia is an increase in cell number.

We have modified our wording to describe our epithelial thickening phenotype as hypertrophic.

The 'flip up' process originally proposed by Coleman in 1965 has been studied many times in recent decades with much support for the hypothesis. These latter studies would be worth citing given they provide some experimental support.

We have included more recent citations describing and supporting the flip up process of palatal shelf elevation.

The studies do not show that both the basal and periderm are required. The K14-Cre indeed shows that the basal epithelia are not sufficient. But the Ect-Cre cannot rule out that only the periderm is required as both are affected. If the authors used a periderm-specific Cre, then they could draw such a definitive conclusion.

We have generated a periderm specific knockout which is now characterized in the manuscript to tease apart the contributions of each layers. We used a Krt6-Cre driver to delete Arhgap29 in the periderm specifically. Our results reveal a surprising and unique role for Arhgap29 in the periderm. These embryos have a fully penetrant primary cleft palate, but no cleft of the secondary palate. Because of this finding, we went back to our other models and scored all previously-analyzed embryos for a primary cleft palate. Both the K14-Cre and the Ect-Cre show some primary cleft, but never a fully penetrant phenotype. We provide extensive description of all these new findings and modified our conclusions to better reflect our data.

Like gene names, the different Cre alleles should also be italicized. The source of the Cre drivers should also be listed or acknowledged.

We have changed the naming of our Cre alleles and listed the source of these drivers.

The Methods sections - Histological analysis and phenotype scoring (first three sentences) and Fluorescent immunostaining and microscopy (first sentence) - need to be rewritten as the grammar is poor.

We have modified the text to improve the grammar and flow. The first sentences of histological analysis now reads “Serial coronal paraffin sections (7µm thick) were obtained and stained with hematoxylin and eosin for histologic analysis. Images were acquired using a Nikon Eclipse E-600 (Nikon, Melville NY) and measurement of palatal epithelium thickness was performed using Nikon NIS-Element software”. The first sentence of immunostaining and microscopy now reads “Immunostaining of paraffin sections was performed as previously described [78]. Four percent paraformaldehyde (PFA) fixed, paraffin embedded slides were de-paraffinized, permeabilized with 0.5% TritonX-PBS and then treated with 0.05% Tweeen-20 citric acid antigen retrieval”.

The authors state that the conditional Arhgap29 animals lose weight following weaning. However, data is only shown (Fig S2) for animals at weaning, and already their weight is low. If weights were taken at other ages then this should be shown. If not, the authors need to change the conclusions - it would seem that the conditional null animals have reduced weight at weaning, but perhaps not through weight loss.

We have modified out conclusions to better reflect what our data really show. The sentence now reads” At weaning, Arhgap29-Ect^{Cre+} homozygous pups exhibit a significant reduction in body weight that continues through adulthood, likely due to an overall reduction in body size.”

It has previously been suggested that ectopic adhesions arise because of changes in E-cadherin (or other adhesion proteins) on the periderm. Are there any quantitative

© 2026. Published by The Company of Biologists under the terms of the Creative Commons Attribution License (https://creativecommons.org/licenses/by/4.0/).

differences in apical E-cadherin staining in mutants vs controls?

No obvious difference in E-cadherin apical localization is observed. Because of this, and the fact that we do not observe ectopic adhesions in our mutants, formal quantification of apical E-cadherin was not performed.

Reviewer 3: SUMMARY OF THE ADVANCE MADE IN THIS PAPER AND ITS POTENTIAL SIGNIFICANCE TO THE FIELD

This manuscript reports analysis of the function of Arhgap29 in palate development using a conditional knockout approach in mice. The authors report that early ectodermal Cre-mediated deletion of Arhgap29, but not K14-Cre mediated oral epithelial specific deletion of Arhgap29, caused a partial but significant penetrance of cleft palate associated with changes in palatal epithelial morphology and expression of markers of cell contractility. The authors conclude this study provides the first direct evidence that ARHGAP29 is essential for proper palatal elevation and fusion.

SUGGESTIONS TO AUTHORS

Several issues need to be resolved to support the authors' conclusions:

1. The title is vague regarding ARHGAP29 function. A more appropriate title could be "ARHGAP29 plays an important role in palatal epithelial organization during palatogenesis".

We have modified our title to better reflect out conclusions. The new title reads: “The ectodermal loss of ARHGAP29 alters epithelial morphology and disrupts murine palatal development”

2. The description and analysis of the Arhgap29 conditional allele are not sufficiently informative. Previous studies suggested Arhgap29 heterozygosity caused oral epithelial adhesions, but no such phenotype is observed in either the K14-Cre cko or the Ect-Cre cko embryos [by the way, the Ect-Cre completely inactivate its function. The manuscript describes the A29fl allele as a FLAG tagged flossed Arhgap29 allele generated at the University of Iowa Core Facility. Figure 1A shows a schematic with loxP sites in introns 3 and 5. It is not clear whether deletion of exons 4 and 5 results in a null allele. There is no description whether germline or early embryonic deletion of the A29fl allele recapitulates the previous A29 homozygous knockout phenotype. Or whether the previous nonsense allele might have a dominant negative effect to account for the oral epithelial adhesion defects observed.

We have corrected the nomenclature of the Cre-drivers and included more information on the alleles used and lethality of previous mouse models. The Arhgap29 floxed allele used in our study was a generous gift from Dr. Cleaver. Arhgap29$^{fl/fl}$-Sox2^{Cre} conditional knockouts are early embryonic lethal similar to the *Arhgap29* null phenotype suggesting that the *Arhgap29* floxed allele does in fact function as a null. The absence of oral adhesions in the conditional knockouts could be due to the timing of cre activation compared to Arhgap29 heterozygotes which lack Arhgap29 from the time of conception.

3. As the manuscript showed, the Ect-Cre has Cre expression/activity in the palatal mesenchyme. The authors indicated that the A29-Ect-cre should be considered a combined mesenchyme knockdown/ectoderm knockout rather than a strict ectoderm-specific conditional murine model. The lack of phenotype of K14-Cre cko together with the presence of significant mesenchymal Cre activity and incomplete penetrance of cleft palate in the Ect-Cre cko mutants raise obvious questions whether Arhgap29 function is required in the palatal epithelium for palate development and whether the variability in cleft palate could primarily be due to the incomplete knockdown in the palatal mesenchyme or other nonpalatal structures. The manuscript subheading of a results section as "ARHGAP29 in oral epithelial cells only is not required for proper palatogenesis" and describing the Ect-Cre cko palate defect as "secondary cleft palate" further confuse the matter. While the data presented provide solid evidence for

© 2026. Published by The Company of Biologists under the terms of the Creative Commons Attribution License (https://creativecommons.org/licenses/by/4.0/).

changes in palatal epithelial morphology, organization, and expression of contractility markers, additional studies are needed to elucidate whether those changes are due to loss of A29 in the palatal epithelium or mesenchyme or both.

We have significantly changed our manuscript to include two additional murine models (periderm-specific knockout and mesenchyme-specific knockout of *Arhgap29*). Further characterization of all murine models reveal the presence of a rare form of primary and/or secondary cleft palate in all epithelial knockout models while no phenotypes were observed when Arhgap29 was deleted specifically in the mesenchyme, suggesting that it is the function of ARHGAP29 in the ectoderm that is primarily required for palatogenesis.

4. The alterations in epithelial morphology, organization, and marker expression are interesting, but whether and how they correlate with cleft palate are not clear. More careful analysis of embryos at E13.5 to E14.5, prior to palatal shelf fusion in both the control and mutant embryos, should be analyzed and scored to see how the changes are consistent with genotypes. If all mutant embryos exhibit consistent epithelial changes, then why only ~23% mutant embryos showed cleft palate?

We agree that how the epithelial defects might contribute to cleft are not clear. We have performed additional analysis of epithelial and contractility defects at earlier timepoints and have modified our text to better reflect our hypothesis of how these defects contribute to cleft.

First decision letter

MS ID#: dev.205636

MS TITLE: The ectodermal loss of ARHGAP29 alters epithelial morphology and disrupts murine palatal development

AUTHORS: Emily Christine Adelizzi, Lindsey Rhea, Campbell Mitvalsky, Samuel Pek, Bethany Dolittle and Martine Dunnwald

Dear Dr Dunnwald,

I have now received all the referees reports on the above manuscript, and have reached a decision. The referees' comments are appended below.

The overall evaluation is positive and we would like to publish a revised manuscript in Development, provided that the referees' comments can be satisfactorily addressed. As you will see two of the revisers have some further minor comments for correction/improvement. Please attend to all of the reviewers' comments in your revised manuscript and detail them in your point-by-point response. If you do not agree with any of their criticisms or suggestions explain clearly why this is so. If it would be helpful, you are welcome to contact us to discuss your revision in greater detail. Please send us a point-by-point response indicating your plans for addressing the referees' comments, and we will look over this and provide further guidance.

Reviewer 1

SUMMARY OF THE ADVANCE MADE IN THIS PAPER AND ITS POTENTIAL SIGNIFICANCE TO THE FIELD

This manuscript reports analysis of the function of Arhgap29 in palate development using a conditional knockout approach in mice. The authors report that early ectodermal Cre-mediated deletion of Arhgap29, but not K14-Cre mediated oral epithelial specific deletion of Arhgap29, caused a partial but significant penetrance of cleft palate associated with changes in palatal epithelial morphology and expression of markers of cell contractility. The authors conclude this

© 2026. Published by The Company of Biologists under the terms of the Creative Commons Attribution License (https://creativecommons.org/licenses/by/4.0/).

study provides the first direct evidence that ARHGAP29 is essential for proper palatal elevation and fusion.

SUGGESTIONS TO AUTHORS

The revised manuscript is much improved. But several mistakes in the text need to be corrected:

1. The manuscript is still unclear and confusing about the Arhgap29fl mice that they used in this study. In the first paragraph in the Results section, it states "we crossed an Arhgap29flox (Arhgap29fl) allele (Xu et al. 2011)..." However, the Xu et al 2011 paper never mentioned any Arhgap29flox allele. The Cleaver lab first cited the Arhgap29fl (KOMP) mice in their Barry et al 2016 paper. If this is the mouse line used, then the correct reference should be Barry et al 2016. Adding to the confusion is that the Methods section says "mice homozygous for the FLAG tagged floxed Arhgap29 allele (Arhgap29fl, generated at teh University of Iowa Core Facility)...", which seems to suggest that this allele is different from the one the Cleaver lab used but doe not provide any information about the structure of the allele and where the FLAG sequence was inserted and where the loxP sites were inserted. If this study indeed used the Arhgap29fl mice from the Cleaver lab, then just cite the source reference in the Methods section but should claim the line was generated at teh University of Iowa Core Facility. On the other hand, if the mouse line is indeed generated at University of Iowa, then the reference citation and the acknowledgement of Dr. Cleaver provided the Arhgap29flox mice would not make sense. Or were two different Arhgap29flox mouse lines used in this study?

2. In the first paragraph in the Results section, Line 7 from bottom of the page, this sentence likely describes Arhgap29-K6Cre+ mice rather than "Arhgap29-K14Cre+" mice because the sentence before this one described a different phenotype in the "Arhgap29fl-K14Cre+" mice. In addition, the citation of Figure 1G-H" is missing.

3. In several places in the manuscript, wherever used, the term "secondary cleft palate" should be changed to "cleft secondary palate", unless the authors meant to say that the cleft palate resulted from a secondary effect. Similarly, several places talked about cleft primary palate as "primary cleft palate", which just creates confusion. Better to use "cleft primary palate" for the primary palate only defect, "cleft secondary palate" for the secondary palate defect, and "cleft primary and secondary palate" for the combined defects.

Reviewer 2

SUMMARY OF THE ADVANCE MADE IN THIS PAPER AND ITS POTENTIAL SIGNIFICANCE TO THE FIELD

This paper shows for the first time that RhoA GTPase Activating Protein 29 (ARHGAP29) is required in the murine palatal epithelium before E14.5 for cytoskeletal organization and drives changes in cell morphology that are important for correct secondary palate development. The authors show that ARHGAP29 negatively regulates expression of contractility markers providing a potential mechanism by which loss of Arhgap29 affects cell morphology in the palate. Arhgap29 is required for elevation and fusion of the palatal shelves- showing for the first time that ARHGAP29 is required for secondary palate development, defining a spatio temporal requirement for arhgap29 in palate development. Because mutations in ARHGAP29 are associated with cleft palate in humans, this work shows that these mutations are, in fact, likely causative for the cleft palate phenotype.

SUGGESTIONS TO AUTHORS

There is one reference that the citation manager did not integrate into the correct formating: Ozekin, 2023 #427#

I also suggest changing the subtitle "Loss of ARHGAP29 promotes the expression of contractility markers in the MEE to " ARHGAP29 supresses expression of contractility markers in the MEE". This helps the reader see what the protein does instead of what happens when it is gone.

Reviewer 3

© 2026. Published by The Company of Biologists under the terms of the Creative Commons Attribution License (https://creativecommons.org/licenses/by/4.0/).

SUMMARY OF THE ADVANCE MADE IN THIS PAPER AND ITS POTENTIAL SIGNIFICANCE TO THE FIELD

Adelizzi et al employ three different epithelial/ectodermal-specific Cre drivers to produce conditional knockout lines of Arhgap29, a gene contributing to risk of cleft lip/palate in humans. Each of the Cre drivers is activated at different times during embryonic facial development and in either the basal epithelia, the periderm, or both. The addition of the periderm-specific conditional mutant has been added for this revised manuscript and has allowed them to better analyze the cell type importance of Arhgap29 in secondary palatal development. They describe the penetrance of different palatal phenotypes in each line, also finding (new to the revision) that there is an anterior secondary palatal defect in some embryos - a defect in the fusion of the anterior aspect of the palatal shelves with the primary palate.

They note oral epithelial changes (hyperplasia) in mutants prefusion, and increased apoptosis during fusion which is not seen in wildtype embryos. These changes in cellular appearance are reflected in the activation or ectopic expression of various molecular markers. They conclude that the palatal phenotype is due to changes in cytoskeletal contractility, which seem resonable, although no direct evidence is provided.

SUGGESTIONS TO AUTHORS

The authors have responded reasonably well to the concerns raised in the initial round of review. In addition, they have added useful new data to address some of those concerns, and extensively rewritten parts of the manuscript. However, numerous issues persist and some new issues have arisen - most of which are fairly minor in the scheme of things. These are listed below.
Despite the request in the prior critique and their response acknowledging this, the authors have not fixed the vast majority of instances where the gene and mouse allele names should have been italicized.
The following sentence (in the latter part of the Introduction) should be broken in two as it is clumsily written: "This single-nucleotide, nonsense mutation produces a truncated protein that is presumed to lack function because no homozygous Arhgap29K326X offspring were observed (as early as E8.5), while heterozygosity for this allele did not result in CL/P, it did cause intra-oral adhesions, a well-accepted hallmark of cleft palate (Antiguas et al., 2022, Ingraham et al., 2006, Kousa et al., 2017).".

In the first Results section, I feel the sentence starting "In Arhgap29-K14Cre+ embryos, a reduction of ARHGAP29 in the periderm was difficult to detect…." should be referring to Arhgap29- K6Cre+, in line with the figure. It should also refer to the appropriate panels in the figure.

In the section "Arhgap29-EctCre+ embryos display gross morphological defects", the authors refer to the homozygous embryos appearing smaller. However, this is not clear in the provided embryo images. Is there some quantification to support this? If not, the wording should perhaps be changed.

Also, the sentence beginning "We identify occasional, yet significant Arhgap29-EctCre+ homozygotes" is poorly worded. The homozygotes are not significant - it is presumably the frequency/incidence of eye defects.

It is appreciated that the authors chose E14.5 as a time point to address the molecular and cellular changes during palatal elevation and fusion. However, given that the genetic background is known to impact the developmental timing of different processes, wouldn't it have been more robust to assess at E15.0 or E15.5 to determine definitive penetrance values of clefting in the different conditional mutants, especially since the authors acknowledge that these conditional lines are of mixed background. Could this mixed background contribute to the different incidence of cleft palate in the mutants? Related to this, the authors state that some of the Arhgap29-EctCre homozygous embryos have unelevated shelves. If the embryos are smaller (ie. delayed) wouldn't it be expected that shelf elevation might also be delayed slightly?

© 2026. Published by The Company of Biologists under the terms of the Creative Commons Attribution License (https://creativecommons.org/licenses/by/4.0/).

In the Discussion, the authors refer to the recently generated CRISPR Arhgap29 KO line, stating that the mutants are viable and display craniofacial defects. The cited papers, however, clearly indicate that these homozygote KOs are not viable. Yes, they survive until late in gestation which is in stark contrast to the original Arhgap29 KO lines reported by the authors, but they are definitely not viable - a term meant for postnatal survival.

The new analysis that has been included by the authors points to a delay, in some of the conditional mutants, of the full fusion of the anterior end of the secondary palatal shelves with the primary palate. It would be helpful to include a full palatal view of one of these defects (in Supplementary data?) rather than being reliant on a cross-sectional histology image, which can vary by position, especially around the incisive foramina. Also, and most importantly, this defect is not a primary palatal cleft. A primary palatal cleft involves a cleft of the alveolus. It is simply an anterior secondary palatal cleft presentation. So all references to a primary palatal cleft need to be removed to avoid misleading readers about the nature of the defect.

The authors state that "one may speculate that there is a temporal requirement for ARHGAP29 in preventing oral adhesions and this [would] (spelling error) occur prior to activation of any of the Cre-drivers used in our studies." This is confusing because the Cre expression in all lines comes on well before fusion, albeit at different times. And, certainly, the Ect-Cre is active in the ectoderm over 5 days before palatal shelf fusion. The authors need to better communicate what they are trying to say here, because - as stated - it does not seem a viable explanation.

Figure 2 - The whole embryo views (side views) are not high enough resolution in the version available to reviewers, but can the authors clarify whether the A29-K6Cre embryo (panel K) has a normal mouth? From the low image resolution, it appears from side on that there may be lateral fusion between the maxilla and mandible, which would result in a small mouth. Such fusions have been occasionally observed in other models.

Figure 4 - In panels A-D, the sections are not from equivalent locations. For example, the wildtype section is middle-to-anterior, whereas the K14Cre and K6Cre sections are posterior sections (as the common nasal passage is evident). Perhaps the wrong molars were used as landmarks to identify the section. This should be fixed/standardized.

Supplemental Fig S1 - Panels H and I are labeled on the figure as primary palate. However, the WT section is clearly not anterior, given the tongue attachment and common nasal passage is still evident. So it is a section from the posterior palate. The legend also needs to be fixed as it refers to these panels as being from the 'middle secondary palate' - which is not the case for either section. Also, for panels K and L, these sections are stated as being at the intersection between the primary and secondary palates, which they are not - based on the nasal septum position. In addition, the WT and mutant sections are remarkably similar in many respects - eg. morphology of each molar, the shape of each ossifying section, gland morphology. It is possible that, just by chance, the near exact orientation and section was chosen in two unrelated embryos. But, it seems more likely that these are serial sections from the same embryo - perhaps inadvertently used. The authors should check this carefully.

First revision

Author response to reviewers' comments

The revised manuscript is much improved. But several mistakes in the text need to be corrected:

The manuscript is still unclear and confusing about the Arhgap29fl mice that they used in this study. In the first paragraph in the Results section, it states "we crossed an Arhgap29flox (Arhgap29fl) allele (Xu et al. 2011)..." However, the Xu et al 2011 paper never mentioned any Arhgap29flox allele. The Cleaver lab first cited the Arhgap29fl (KOMP) mice in their Barry et al 2016 paper. If this is the mouse line used, then the correct reference should be Barry et al

© 2026. Published by The Company of Biologists under the terms of the Creative Commons Attribution License (https://creativecommons.org/licenses/by/4.0/).

2016. Adding to the confusion is that the Methods section says "mice homozygous for the FLAG tagged floxed Arhgap29 allele (Arhgap29fl, generated at teh University of Iowa Core Facility)...", which seems to suggest that this allele is different from the one the Cleaver lab used but does not provide any information about the structure of the allele and where the FLAG sequence was inserted and where the loxP sites were inserted. If this study indeed used the Arhgap29fl mice from the Cleaver lab, then just cite the source reference in the Methods section but should claim the line was generated at teh University of Iowa Core Facility. On the other hand, if the mouse line is indeed generated at University of Iowa, then the reference citation and the acknowledgement of Dr. Cleaver provided the Arhgap29flox mice would not make sense. Or were two different Arhgap29flox mouse lines used in this study?

The reviewer is correct, two different *Arhgap29* floxed alleles were used in this study. One is the same floxed allele described in Barry et al (generous gift from Dr. Cleaver), and the other is one that we generated by adding a FLAG tag to the already available floxed allele. We clarified the description of the *Arhgap29* floxed alleles used in the main text and methods, updated the schematics in our figures and supplement to clarify these differences, and made proper reference to publications when appropriate.

In the first paragraph in the Results section, Line 7 from bottom of the page, this sentence likely describes Arhgap29-K6Cre+ mice rather than "Arhgap29-K14Cre+" mice because the sentence before this one described a different phenotype in the "Arhgap29fl-K14Cre+" mice. In addition, the citation of Figure 1G-H" is missing.

Thank you for pointing this out. We corrected the text to include the correct mouse model and a citation for Figure 1G-H.

In several places in the manuscript, wherever used, the term "secondary cleft palate" should be changed to "cleft secondary palate", unless the authors meant to say that the cleft palate resulted from a secondary effect. Similarly, several places talked about cleft primary palate as "primary cleft palate", which just creates confusion. Better to use "cleft primary palate" for the primary palate only defect, "cleft secondary palate" for the secondary palate defect, and "cleft primary and secondary palate" for the combined defects.

We agree with the reviewer that descriptions of the cleft phenotypes were confusing, and actually not correct (see answer to reviewer 3). We have modified our text to include proper nomenclature.

Reviewer 2:

There is one reference that the citation manager did not integrate into the correct formating: Ozekin, 2023 #427#

We corrected the formatting for this reference in the text.

I also suggest changing the subtitle "Loss of ARHGAP29 promotes the expression of contractility markers in the MEE to " ARHGAP29 suppresses expression of contractility markers in the MEE ". This helps the reader see what the protein does instead of what happens when it is gone.

We thank the reviewer for their suggestion and modified the text to include the revised title
"ARHGAP29 suppresses markers of contractility in the MEE"

Reviewer 3:

Despite the request in the prior critique and their response acknowledging this, the authors have not fixed the vast majority of instances where the gene and mouse allele names should have been italicized.

We systematically reviewed our manuscript for nomenclature where the gene and mouse allele names should have been italicized. We corrected this issue to the best of our knowledge. We found that in many instances, the sentence could read either with the gene or the protein in mind. For example, in the abstract, we say that "yet the role and tissue-specific requirements for ARHGAP29 during palatogenesis is unknown." In this case, it relates to both the gene and the protein, and we chose to refer to the protein as demonstrated by the uppercase

© 2026. Published by The Company of Biologists under the terms of the Creative Commons Attribution License (https://creativecommons.org/licenses/by/4.0/).

spelling. We would welcome suggestions from this reviewer if issues remain in the current submitted manuscript.

The following sentence (in the latter part of the Introduction) should be broken in two as it is clumsily written: "This single-nucleotide, nonsense mutation produces a truncated protein that is presumed to lack function because no homozygous Arhgap29K326X offspring were observed (as early as E8.5), while heterozygosity for this allele did not result in CL/P, it did cause intra-oral adhesions, a well-accepted hallmark of cleft palate (Antiguas et al., 2022, Ingraham et al., 2006, Kousa et al., 2017)."

We modified the text to improve the flow of this sentence.

In the first Results section, I feel the sentence starting "In Arhgap29-K14Cre+ embryos, a reduction of ARHGAP29 in the periderm was difficult to detect…." should be referring to Arhgap29- K6Cre+, in line with the figure. It should also refer to the appropriate panels in the figure.

We corrected the text to include the correct mouse model.

In the section "Arhgap29-EctCre+ embryos display gross morphological defects", the authors refer to the homozygous embryos appearing smaller. However, this is not clear in the provided embryo images. Is there some quantification to support this? If not, the wording should perhaps be changed.

We removed the statement about the size of the embryo as we have no formal quantification for the size of the embryos (we do for postnatal animals and this is provided in supplemental figure 3).

Also, the sentence beginning "We identify occasional, yet significant Arhgap29-EctCre+ homozygotes" is poorly worded. The homozygotes are not significant - it is presumably the frequency/incidence of eye defects.

We modified the text to clarify this sentence.

It is appreciated that the authors chose E14.5 as a time point to address the molecular and cellular changes during palatal elevation and fusion. However, given that the genetic background is known to impact the developmental timing of different processes, wouldn't it have been more robust to assess at E15.0 or E15.5 to determine definitive penetrance values of clefting in the different conditional mutants, especially since the authors acknowledge that these conditional lines are of mixed background. Could this mixed background contribute to the different incidence of cleft palate in the mutants?

We appreciate these comments from the reviewer and agree that there are variations in the timing of palatogenesis and incidence of cleft based on genetic background. We designed our study to detect potential delays at E14.5 compared to littermate controls and rather than using E15.5 to determine definitive penetrance we chose E18.5 which is even closer to birth and less likely to resolve. The reviewer makes a good point on background contribution. While our mice are of a mixed background, we expect the Arhgap29-K14^{Cre+} and Arhgap29-Ect^{Cre+} to be more similar as they are primarily C57. As the Arhgap29-K6^{Cre+} was recently added to our colony it is possible the background could contribute to the higher anterior cleft incidence and we have included a brief mention of this in the second to last paragraph of our discussion.

Related to this, the authors state that some of the Arhgap29-EctCre homozygous embryos have unelevated shelves. If the embryos are smaller (ie. delayed) wouldn't it be expected that shelf elevation might also be delayed slightly?

We don't believe that the observed reduced weight means that embryos are solely delayed in their development given that we see this reduced weight through adulthood. We would expect that if the defects we observe are the result of a slight delay in palatogenesis that this would not result in a cleft phenotype at E18.5.

In the Discussion, the authors refer to the recently generated CRISPR Arhgap29 KO line, stating that the mutants are viable and display craniofacial defects. The cited papers, however, clearly indicate that these homozygote KOs are not viable. Yes, they survive until late in gestation

© 2026. Published by The Company of Biologists under the terms of the Creative Commons Attribution License (https://creativecommons.org/licenses/by/4.0/).

which is in stark contrast to the original Arhgap29 KO lines reported by the authors, but they are definitely not viable - a term meant for postnatal survival.

We modified our writing to more accurately describe the survival of Arhgap29 CRIPSR KO.

The new analysis that has been included by the authors points to a delay, in some of the conditional mutants, of the full fusion of the anterior end of the secondary palatal shelves with the primary palate. It would be helpful to include a full palatal view of one of these defects (in Supplementary data?) rather than being reliant on a cross-sectional histology image, which can vary by position, especially around the incisive foramina. Also, and most importantly, this defect is not a primary palatal cleft. A primary palatal cleft involves a cleft of the alveolus. It is simply an anterior secondary palatal cleft presentation. So all references to a primary palatal cleft need to be removed to avoid misleading readers about the nature of the defect.

We added a full palatal view of the anterior cleft to corroborate the histological findings (Supplemental figure 4). We also thank the reviewer for pointing out that this cleft is not a cleft of the primary palate, but rather a cleft between the most anterior region of the secondary palate and the primary palate. We modified the description of the observed phenotype in the text to properly describe this anterior cleft palate defect.

The authors state that "one may speculate that there is a temporal requirement for ARHGAP29 in preventing oral adhesions and this [would] (spelling error) occur prior to activation of any of the Cre- drivers used in our studies." This is confusing because the Cre expression in all lines comes on well before fusion, albeit at different times. And, certainly, the Ect-Cre is active in the ectoderm over 5 days before palatal shelf fusion. The authors need to better communicate what they are trying to say here, because - as stated - it does not seem a viable explanation.

Our sentence refers to <u>oral adhesions</u>, which are abnormal tissue-tissue adhesions occurring in the oral cavity of some genetically modified animals (i.e. *$Arhgap29^{K326X}$* heterozygotes, *$Irf6^{nl}$* heterozygotes, etc). They are thought to arise when the periderm in the oral cavity is absent or malfunctioning and have been considered a hallmark for CLP. These adhesions were reported as early as E12.5, and if present are most abundant at earlier timepoints E13.5 and E14.5. We added an example to more clearly indicate what we are referencing in our discussion.

Figure 2 - The whole embryo views (side views) are not high enough resolution in the version available to reviewers, but can the authors clarify whether the A29-K6Cre embryo (panel K) has a normal mouth? From the low image resolution, it appears from side on that there may be lateral fusion between the maxilla and mandible, which would result in a small mouth. Such fusions have been occasionally observed in other models.

We do not observe any macroscopic craniofacial abnormalities or small mouths in our Arhgap29-K14Cre+ or Arhgap29-K6Cre+ embryos, rather there are slight changes and variability of appearance due to the angle of the embryo when the image is acquired. We added a specific comment on the lack of macroscopic craniofacial abnormalities in our description of these embryos in the text of the result section.

Figure 4 - In panels A-D, the sections are not from equivalent locations. For example, the wildtype section is middle-to-anterior, whereas the K14Cre and K6Cre sections are posterior sections (as the common nasal passage is evident). Perhaps the wrong molars were used as landmarks to identify the section. This should be fixed/standardized.
We agree with the reviewers that our current images are not at equivalent locations. We changed the panels such that the new images are all from the same anatomical locations.

We agree with the reviewer that panels A-D are not from equivalent locations. We have replaced the representative images for Arhgap29-$K14^{Cre+}$ and Arhgap29-$K6^{Cre+}$ with images in similar locations as the wild-type and Arhgap29-Ect^{Cre+}.

Supplemental Fig S1 - Panels H and I are labeled on the figure as primary palate. However, the WT section is clearly not anterior, given the tongue attachment and common nasal passage is

© 2026. Published by The Company of Biologists under the terms of the Creative Commons Attribution License (https://creativecommons.org/licenses/by/4.0/).

still evident. So it is a section from the posterior palate. The legend also needs to be fixed as it refers to these panels as being from the 'middle secondary palate' - which is not the case for either section. Also, for panels K and L, these sections are stated as being at the intersection between the primary and secondary palates, which they are not - based on the nasal septum position. In addition, the WT and mutant sections are remarkably similar in many respects - eg. morphology of each molar, the shape of each ossifying section, gland morphology. It is possible that, just by chance, the near exact orientation and section was chosen in two unrelated embryos. But, it seems more likely that these are serial sections from the same embryo - perhaps inadvertently used. The authors should check this carefully.

> **We agree with the reviewers that panels H and I are not depicting the primary palate and corrected our description in the text, figures, and methods and included the proper images. We also corrected our description of the location of images M and N. We thank the reviewer for their careful analysis of our histology and confirmed that images M and N were taken from two different embryos and not consecutive sections of the same embryo.**

Second decision letter

MS ID#: dev.205636R1

MS TITLE: The ectodermal loss of ARHGAP29 alters epithelial morphology and disrupts murine palatogenesis

AUTHORS: Emily Christine Adelizzi, Lindsey Rhea, Campbell Mitvalsky, Samuel Pek, Bethany Dolittle and Martine Dunnwald

Dear Dr Dunnwald,

I have now received all the referees reports on the above manuscript, and have reached a decision. The referees' comments are appended below.

The overall evaluation is positive and we would like to publish a revised manuscript in Development, provided that Reviewer 3 comments can be satisfactorily addressed. Please attend to their comments in your revised manuscript and detail them in your point-by-point response. If you do not agree with any of their criticisms or suggestions explain clearly why this is so. As the suggestions are minor the revised manuscript will not require further review, rather I will look it over prior to acceptance.

Reviewer 1

SUMMARY OF THE ADVANCE MADE IN THIS PAPER AND ITS POTENTIAL SIGNIFICANCE TO THE FIELD

No additional comments

Comments for the author

The revised manuscript has addressed my comments. I have no additional comments

Reviewer 3

SUMMARY OF THE ADVANCE MADE IN THIS PAPER AND ITS POTENTIAL SIGNIFICANCE TO THE FIELD

The authors report the use of different ectodermal Cre drivers ("basal", periderm, and both) to further investigate the role of Arhgap29 in palatogenesis, after previously reporting that ubiquitous

© 2026. Published by The Company of Biologists under the terms of the Creative Commons Attribution License (https://creativecommons.org/licenses/by/4.0/).

knockout Arhgap29 mice exhibit ectopic oral epithelial adhesions. In this manuscript, they report the incidence of palatal anomalies (cleft palate and failed fusion between the primary and secondary palates) in the different conditional mutants and characterize molecular and cellular changes associated with these phenotypes. This second-time revised manuscript again addresses many of the remaining concerns of the last review, although some minor issues still persist.

SUGGESTIONS TO AUTHORS

The authors have largely addressed many remaining concerns, although it still appears that reference to 'alleles' (ie. when to use italics) cannot be recognized by the authors. To assist with this, whenever there is a reference to mice that carry the floxed alleles and/or the Cre, these should be italicized: for example, Arhgap29-Wnt1Cre+, Arhgap29-K14Cre+, Arhgap29-K6Cre+, and Arhgap29-EctCre+ as they are all referred to by the combination of ALLELES. Furthermore, in all cases, the Arhgap29 allele should be referred to in full, eg. Arhgap29fl/fl, not shortened to just Arhgap29. This also clarifies to the reader that the animals are homozygous for the floxed allele, not heterozygous.

In the section: Arhgap29-EctCre+ embryos display gross morphological defects, please correct 'occular' to 'ocular'

Although the authors have replaced all "A29" with "Arhgap29" throughout the main text, All figure legends still contain this inappropriate abbreviation.

In the Discussion, the sentence "However, a recent publication reported the generation of an Arhgap29 knockout mouse using CRISPR (Chi et al., 2025; Zhang et al., 2025)" provides no context for the 'however'. To do so, the authors should reword this sentence to combine it with the sentence that follows it. A suggestion might be: However, contrary to these earlier findings, a recent publication reported the generation of an Arhgap29 knockout mouse using CRISPR, which survive through gestation (Chi et al., 2025; Zhang et al., 2025). Although these mutants display craniofacial defects, they do not enable the tissue-specific roles of ARHGAP29 to be investigated.

Finally, the authors still do not really discuss or mention the breadth of explanation for the disparate presentations. They do now mention genetic background differences as one possibility, and/or perhaps a temporal requirement for Arhgap29. But another possibility is incomplete excision by the Cre enzyme and/or the spatiotemporal differences in their expression. Also, while the increased expression of contractility markers and increased apoptosis is interesting, the discussion lacks detail (even speculative) on how this could lead to the phenotypes (as well as the ectopic oral adhesions seen previously in the full knockout) without associated changes in adhesion protein levels of localization.

Bottom of the second page of the Discussion, fix the typo: "oral adhesionso" to "oral adhesions".

Second revision

Author response to reviewers' comments

Responses to Reviewer 3:

The authors have largely addressed many remaining concerns, although it still appears that reference to 'alleles' (ie. when to use italics) cannot be recognized by the authors. To assist with this, whenever there is a reference to mice that carry the floxed alleles and/or the Cre, these should be italicized: for example, Arhgap29-Wnt1Cre+, Arhgap29-K14Cre+, Arhgap29-K6Cre+, and Arhgap29-EctCre+ as they are all referred to by the combination of ALLELES. Furthermore, in all cases, the Arhgap29 allele should be referred to in full, eg. Arhgap29fl/fl, not shortened to just Arhgap29. This also clarifies to the reader that the animals are homozygous for the floxed allele, not heterozygous.

© 2026. Published by The Company of Biologists under the terms of the Creative Commons Attribution License (https://creativecommons.org/licenses/by/4.0/).

Thank you to the reviewer for their assistance. We have italicized all the alleles throughout all the documents.

In the section: Arhgap29-EctCre+ embryos display gross morphological defects, please correct 'occular' to 'ocular'

We corrected this in our revised manuscript.

Although the authors have replaced all "A29" with "Arhgap29" throughout the main text, all figure legends still contain this inappropriate abbreviation.

We corrected this in our revised manuscript.

In the Discussion, the sentence "However, a recent publication reported the generation of an Arhgap29 knockout mouse using CRISPR (Chi et al., 2025; Zhang et al., 2025)" provides no context for the 'however'. To do so, the authors should reword this sentence to combine it with the sentence that follows it. A suggestion might be: However, contrary to these earlier findings, a recent publication reported the generation of an Arhgap29 knockout mouse using CRISPR, which survive through gestation (Chi et al., 2025; Zhang et al., 2025). Although these mutants display craniofacial defects, they do not enable the tissue-specific roles of ARHGAP29 to be investigated.

We revised our manuscript according to the reviewer's suggestion.

Finally, the authors still do not really discuss or mention the breadth of explanation for the disparate presentations. They do now mention genetic background differences as one possibility, and/or perhaps a temporal requirement for Arhgap29. But another possibility is incomplete excision by the Cre enzyme and/or the spatiotemporal differences in their expression. Also, while the increased expression of contractility markers and increased apoptosis is interesting, the discussion lacks detail (even speculative) on how this could lead to the phenotypes (as well as the ectopic oral adhesions seen previously in the full knockout) without associated changes in adhesion protein levels of localization.

We provide additional discussion points in our revised manuscript (spatial and temporal regulation of Cre enzyme, incomplete excision) and added a paragraph where we speculate on the role of increased apoptosis in our mutant.

Bottom of the second page of the Discussion, fix the typo: "oral adhesionso" to "oral adhesions".

We looked for this typo and could not find it in our previous version. We scanned our manuscript and believe we spelled all "oral adhesions" correctly.

Third decision letter

MS ID#: dev.205636R2

MS TITLE: The ectodermal loss of ARHGAP29 alters epithelial morphology and disrupts murine palatogenesis

AUTHORS: Emily Christine Adelizzi, Lindsey Rhea, Campbell Mitvalsky, Samuel Pek, Bethany Dolittle and Martine Dunnwald

Dear Dr Dunnwald,

© 2026. Published by The Company of Biologists under the terms of the Creative Commons Attribution License (https://creativecommons.org/licenses/by/4.0/).

I am happy to tell you that your manuscript has been accepted for publication in Development, pending our standard publication integrity checks.

© 2026. Published by The Company of Biologists under the terms of the Creative Commons Attribution License (https://creativecommons.org/licenses/by/4.0/).
